# Systemically engineering *Bacillus amyloliquefaciens* for increasing its antifungal activity and green antifungal lipopeptides production

**DOI:** 10.3389/fbioe.2022.961535

**Published:** 2022-09-07

**Authors:** Susheng Wang, Rui Wang, Xiuyun Zhao, Gaoqiang Ma, Na Liu, Yuqing Zheng, Jun Tan, Gaofu Qi

**Affiliations:** ^1^ College of Life Science and Technology, Huazhong Agricultural University, Wuhan, Hubei, China; ^2^ Enshi Tobacco Technology Center, Enshi City, Hubei, China

**Keywords:** Bacillus amyloliquefaciens, metabolic engineering, lipopeptide, iturin, fengycin

## Abstract

The biosynthesis of antifungal lipopeptides iturin and fengycin has attracted broad interest; however, there is a bottleneck in its low yield in wild strains. Because the key metabolic mechanisms in the lipopeptides synthesis pathway remain unclear, genetic engineering approaches are all ending up with a single or a few gene modifications. The aim of this study is to develop a systematic engineering approach to improve the antifungal activity and biosynthesis of iturin and fengycin in *Bacillus amyloliquefaciens*. First, blocking the carbon overflow metabolic pathway to increase precursor supply of the branched-chain amino acids by knockout of *bdh*, disrupting sporulation to extend the stage for producing antifungal lipopeptides by deletion of *kinA*, blocking of siderophore synthesis to enhance the availability of amino acids and fatty acids by deletion of *dhbF*, and increasing Spo0A∼P by deletion of *rapA*, could improve the antifungal activity by 24%, 10%, 13% and 18%, respectively. Second, the double knockout strain Δ*bdh*Δ*kinA*, triple knockout strain Δ*bdh*Δ*kinA*Δ*dhbF* and quadruple knockout strain Δ*kinA*Δ*bdh*Δ*dhbF*Δ*rapA* could improve the antifungal activity by 38%, 44% and 53%, respectively. Finally, overexpression of *sfp* in Δ*kinA*Δ*bdh*Δ*dhbF*Δ*rapA* further increased the antifungal activity by 65%. After purifying iturin and fengycin as standards for quantitative analysis of lipopeptides, we found the iturin titer was 17.0 mg/L in the final engineered strain, which was 3.2-fold of the original strain. After fermentation optimization, the titer of iturin and fengycin reached 31.1 mg/L and 175.3 mg/L in flask, and 123.5 mg/L and 1200.8 mg/L in bioreactor. Compared to the original strain, the iturin and fengycin titer in bioreactor increased by 22.8-fold and 15.9-fold in the final engineered strain, respectively. This study may pave the way for the commercial production of green antifungal lipopeptides, and is also favorable for understanding the regulatory and biosynthetic mechanism of iturin and fengycin.

## Introduction

**FIGURE 1:**
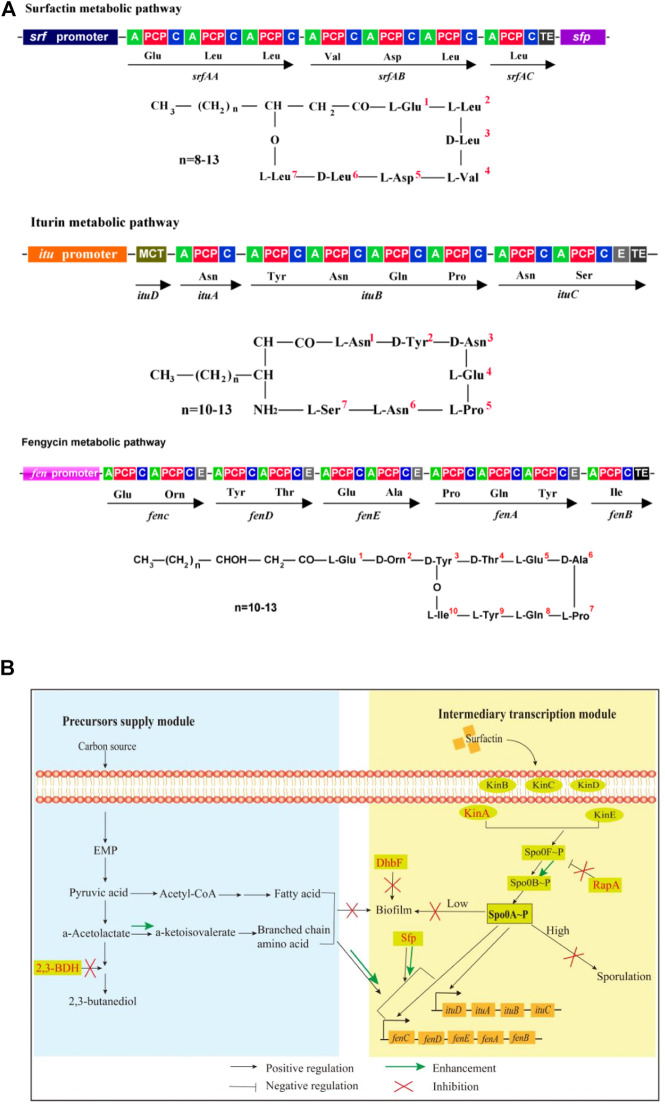
Molecular structures, biosynthesis pathways and regulator network of lipopeptides. **(A)**: Molecule structures and biosynthesis pathways of lipopeptides. In the figures, “A” means amino acid activating domain; “PCP” means peptidyl carrier protein; “C” means condensation domain; “E” means epimerization domain; “TE” means thioesterase domain; “MCT” means monocarboxylate transporter. **(B)**: Regulatory network for biosynthesis of iturin and fengycin. 2,3-BDH: 2,3-butanediol dehydrogenase; Kin A-E: histidine kinases; DhbF: biosynthesis of siderophore; Spo0A: global regulator; Spo0F and Spo0B: phosphate group transporter; RapA: aspartate phosphatase; Sfp:4-phosphopantetheinyl transferase.

Many *Bacillus* species such as *B. amyloliquefaciens*, *B. subtilis*, *B. velezensis*, etc, are well-known bacteria for producing antifungal lipopeptides such as iturin and fengycin (Kaspar et al., 2019), which are green and broad-spectrum fungicides with potential use in developing biological pesticides (Zhao et al., 2017b), food preservatives (Kourmentza et al., 2021; Gu et al., 2022), antifungal and antitumor drugs (Lin et al., 2020; Wan et al., 2021), feed additives (Zhao et al., 2017a; Prathiviraj et al., 2021), etc. Iturin, a cyclic lipoheptapeptide that is linked to a fatty acid chain with 14–17 carbon number, has a broad-spectrum inhibitory effect on many plant pathogenic fungi. Recently, it has also been reported with anticancer activities (Wan et al., 2021). Fengycin is a cyclic antimicrobial lipopeptide consisting of a β-hydroxy fatty acid with 13–19 carbon number, which has strong wide-spectrum antifungal and antiviral activities, as well as potential anticancer activity (Gao et al., 2022). Additionally, *Bacillus* can also produce surfactin, a cyclic lipopeptide containing a fatty acid tail and a heptapeptide, as a signal molecule to trigger quorum sensing response (e.g., biofilm formation and sporulation) (Vlamakis et al., 2013; Oslizlo et al., 2014). The structure of lipopeptides is shown in Figure 1A.

Biosynthesis of iturin and fengycin has attracted significant attention in recent years (Yang et al., 2020; Wan et al., 2021). However, low fermentation yield greatly restricts their practical applications and further functional investigations (Yang et al., 2020). Moreover, there have been no reports of the chemical synthesis of iturin and fengycin. Thus, industrial production of the high-value added chemicals iturin and fengycin must rely on bacterial fermentation (Horsburgh and Moir, 1999; Gao et al., 2022). For this purpose, genetic engineering methods should be used to enhance their biosynthesis. Major concerns, however, are attributed to large genetic sequence of the operons encoding iturin (*itu*, 38-kb) and fengycin (*fen*, 30-kb) synthase (Tosato et al., 1997; Wu et al., 2007; Cheng et al., 2017; Yang et al., 2020; Wan et al., 2021). Because direct overexpression of *itu* and *fen* operons has been challenging, *Bacillus* species have been engineered to improve iturin and fengycin yield mainly through promoter exchanges of the synthase operon (Dang et al., 2019), strengthening biosynthesis of the substrates such as fatty acids (He et al., 2021; Tan et al., 2022), overexpression of genes encoding the regulators ComA, SigA, DegU, DegQ and Spo0A (Koumoutsi et al., 2007; Wang et al., 2015; Zhang et al., 2017; Klausmann et al., 2021; Sun et al., 2021), or deletion of the repressors gene such as *abrB* (Xu et al., 2020). These studies are able to efficiently increase the lipopeptides biosynthesis, and also demonstrate the importance of understanding the biosynthetic metabolism of iturin and fengycin. However, these genetic engineering methods can only result in a single or a few gene modifications, and the commercial production of antifungal lipopeptides has still not been achieved. Therefore, knowledge-based optimizations are still ongoing, and global antifungal lipopeptides biosynthesis and regulatory features still need to be explored.

For biosynthesis of lipopeptides, the first is the supply of pyruvate through the glycolytic pathway. Pyruvate can be converted to the branched-chain amino acids, which are precursors for biosynthesis of several lipopeptides such as surfactin, iturin and fengycin. However, pyruvate can also be converted to acetolactate by acetolactate synthase. Subsequently, acetolactate is decarboxylated to acetoin by acetolactate decarboxylase, then acetoin is conversed to 2,3-butanediol by 2,3-butanediol dehydrogenase (2,3-BDH) (Peng et al., 2019). The Sfp protein (4-phosphopantetheinyl transferase) plays an essential role in activation of lipopeptides synthesis by transferring the 4′-phosphopantetheinyl moiety of coenzyme A to a serine residue (Reuter et al., 1999; Wu et al., 2019). The second is lipopeptides assembly catalyzed by the iturin and fengycin synthase, which are encoded by the *itu* and *fen* operons, respectively.

There are several regulators to directly or indirectly regulate the biosynthesis of lipopeptides in *Bacillus* species. DegQ, a small regulatory protein, is positive for production of iturin (Tsuge et al., 2005). The global regulator Spo0A that can be activated to be phosphorylated by the histidine kinases such as KinA and dephosphorylated by the aspartate phosphatases RapA and Spo0E is also essential for production of lipopeptides such as iturin and fengycin (Rahman et al., 2006; Sun et al., 2021). Some regulators for biofilm formation are also involved in biosynthesis of lipopeptides. For example, SinI is negative while SinR is positive for biosynthesis of lipopeptides (Wu et al., 2019). Fur is a regulator to suppress biofilm formation in *B. subtilis* (Pi and Helmann 2017). Both ComA and SigA play a positive role in biosynthesis of lipopeptides (Zhang et al., 2017; Sun et al., 2021). However, AbrB represses the transcription of *itu* operon (Xu et al., 2020). CodY suppresses the biosynthesis of branched-chain amino acids, which are substrates for biosynthesis of lipopeptides (Fujita 2009). Spx and PerR, transcriptional regulatory proteins for redox reaction, regulate the transcription of *srf* operon for biosynthesis of surfactin (Ohsawa et al., 2006).

In our previous work, *B. amyloliquefaciens* WH1 was isolated with excellent antifungal activity from rice root (Supplementary Figure S1). Fortunately, WH1 is easy to be transformed with DNA (Zhang et al., 2022). Here, we used WH1 as an initial host to construct antifungal lipopeptides hyperproducers through system metabolic engineering strategy (Figure 1B). First, we knocked out the *bdh* gene to block biosynthesis of 2,3-butanediol, which could improve production of precursors (branched-chain amino acids). Second, we deleted the *kinA* gene to hinder sporulation, which could extend the stage for production of antifungal lipopeptides (Rahman et al., 2006). Third, we knocked out the *dhbF* gene to disrupt siderophore production for improving biosynthesis of antifungal lipopeptides. Fourth, the gene *rapA* was deleted for maintaining the level of Spo0A∼P, which was essential for biosynthesis of antifungal lipopeptides. Finally, we engineered *sfp* to strengthen the activation of precursors. This work will not only shed new light on biosynthetic and regulatory mechanisms of efficient iturin and fengycin production, but will also increase these two lipopeptides titers in *B. amyloliquefaciens*.

## Materials and methods

### Bacterial strains and materials

Experiments were performed with the strains listed in Table 1. Materials for DNA manipulation were purchased from Takara Bio (China). Other chemicals were of analytical grade supplied by Sinopharm Chemical Reagent (China).

**TABLE 1:** Bacterial strains used in this study.

Strains	Characteristics	Source
*Fusarium oxysporum*	Pathogenic fungus	Stored in this lab
*Bacillus amyloliquefaciens* WH1	Wild-type strain	Stored in this lab Chen et al. (2020)
Δ*ituB*	*ituB* knockout strain	Stored in this lab Chen et al. (2020)
Δ*fenA*	*fenA* knockout strain	Stored in this lab Chen et al. (2020)
Δ*ituB*Δ*fenA*	*ituB* and *fenA* double knockout strain	This study
Δ*kinA*	*kinA* knockout strain	Stored in this lab Chen et al. (2020)
Δ*bdh*	*bdh* knockout strain	This study
Δ*degS*	*degS* knockout strain	This study
Δ*tnrA*	*tnrA* knockout strain	This study
Δ*codY*	*codY* knockout strain	This study
Δ*spo0E*	*spo0E* knockout strain	This study
Δ*rapA*	*rapA* knockout strain	This study
Δ*dhbF*	*dhbF* knockout strain	This study
Δ*fur*	*fur* knockout strain	This study
Δ*abrB*	*abrB* knockout strain	This study
Δ*sinI*	*sinI* knockout strain	Stored in this lab Zhang et al. (2022)
Δ*sinR*	*sinR* knockout strain	Stored in this lab Zhang et al. (2022)
Δ*spo0A*	*spo0A* knockout strain	Stored in this lab Chen et al. (2020)
Δ*spo0A/*T2-*spo0A*	*spo0A-*complementary strain	This study
WH1/T2-*spo0A*	Overexpression of *spo0A* in WH1	This study
Δ*sfp*	*sfp* knockout strain	This study
Δ*kinA*Δ*bdh*	*kinA* and *bdh* double knockout strain	This study
Δ*sfp/*T2-*sfp*	*sfp-*complementary strain	This study
WH1*/*T2-*sfp*	Overexpression of *sfp* in WH1	This study
Δ*codY*Δ*kinA*	*kinA* and *codY* double knockout strain	This study
Δ*tnrA*Δ*kinA*	*kinA* and *tnrA* double knockout strain	This study
Δ*kinA*Δ*bdh*Δ*dhbF*	*kinA*, *bdh* and *dhbF* triple knockout strain	This study
Δ*kinA*Δ*bdh*Δ*dhbF*Δ*spo0E*	*kinA*, *bdh*, *dhbF* and *spo0E* quadruple knockout strain	This study
Δ*kinA*Δ*bdh*Δ*dhbF*Δ*rapA*	*kinA*, *bdh*, *dhbF* and *rapA* quadruple knockout strain	This study
Δ*kinA*Δ*bdh*Δ*rapA*Δ*dhbF/*T2-*sfp*	Overexpression of *sfp* in Δ*kinA*Δ*bdh*Δ*rapA*Δ*dhbF*	This study
Δ*kinA*Δ*bdh*Δ*rapA*Δ*dhbF*Δ*spo0A*	*kinA*, *bdh*, *dhbF*, *rapA* and *spo0A* penta knockout strain	This study
Δ*kinA*Δ*bdh*Δ*rapA*Δ*dhbF*Δ*spo0A/*T2-*spo0A*	Compensation of *spo0A* in Δ*kinA*Δ*bdh*Δ*rapA*Δ*dhbF*Δ*spo0A*	This study

### Mutation of lipopeptides synthase operon

On the basis of the mutant strains Δ*ituB* and Δ*fenA* (Chen et al., 2020), we constructed the *ituB* and *fenA* double mutant strain Δ*ituB*Δ*fenA* (Table 1). The strains WH1, Δ*ituB*, Δ*fenA* and Δ*ituB*Δ*fenA* were determined for the antifungal activity against *Fusarium oxysporum*. Briefly, *F. oxysporum* was inoculated on the center, and WH1, Δ*ituB*, Δ*fenA* and Δ*ituB*Δ*fenA* were inoculated on the right, left, bottom and top of the PDA (potato dextrose agar) plates, respectively. The plates were incubated at 28°C for 3 days, then the antifungal activity was observed.

### Construction of knockout, complementary and overexpression strains

The genes including *bdh, comK*, *sigD*, *tnrA*, *codY*, *spo0E*, *rapA*, *dhbF*, *fur*, *abrB* and *sfp* were deleted by double crossover homologous recombination for constructing the single, double, triple, quadruple and penta knockout strains, respectively (Qi et al., 2014). The detailed methods to construct knockout strains were described in the Supplementary Materials.

The plasmids were constructed for expression of *spo0A* and *sfp*, respectively. Briefly, the genes *spo0A* and *sfp* with their own promoters and terminators were amplified from the genomic DNA of WH1 by PCR with the primers listed in Supplementary Table S1, cloned into the T2 plasmid joined by *BamH* I and *Xba* I restriction sites, then the constructed plasmids were used for transformation of the related hosts, respectively (Qi et al., 2014).

### Determining cell growth and sporulation

The single colony of strains was used for inoculating LB medium and cultured at 37°C overnight, then 2 μl broth was transferred into 200 μl fresh LB medium in 96-well microplates for incubation at 37°C for 48 h. In this period, the growth curves were determined with an automatic growth curve analyzer (Bioscreen Cpro, OY Growth Curves, Finland). The broths were also collected for detecting sporulation via crystal violet staining after being cultured for 48 h (Chen et al., 2020). We also determined the rate of surviving cells after heat treatment. Briefly, after being cultured for 48 h, the broths were heated at 80°C for 10 min in the water bath, used for spreading LB agar plates after serial dilutions, then incubated at 37°C for 24 h. The broths without heating were used for spreading plates as control. The colony numbers were counted for calculating the rate of surviving cells after heating at 80°C for 10 min.

### Detecting biofilm formation

Strains were cultured on LB agar plates, then the morphology of colonies were observed by microscope. Robust pellicles (floating biofilms) were determined in multiwell (24-well) plates (Müller et al., 2015). Briefly, 20 μL fresh broth of each strain was used for inoculating 2 ml MSgg medium in each well, then cultured at 28°C for 48 h to allow float biofilms formation. The MSgg medium contains 100 mM MOPS, 0.5% (v/v) glycerol, 0.5% (w/v) sodium glutamate, 5 mM K_2_SO_4_ (pH = 7), 50 μg/mL L- tryptophan, 50 μg/mL L-Phenylalanine, 2 mM MgCl_2_, 700 μM CaCl_2_, 50 μM FeCl_3_, 50 μM MnCl_2_, 2 µM thiamine and 1 μM ZnCl_2_.

### qRT-PCR

The transcription of *ywaA* and *leuA* was analyzed by qRT-PCR. Single colony of each strain was selected for inoculating LB medium and cultured at 37°C overnight, then the broth was transferred into fresh LB medium at a ratio of 1% (v/v). After incubation at 37°C for 24 h, the broth was collected for isolating mRNA with RNeasy Mini Kit (Qiagen, German). cDNA was produced by reverse transcription with 1 μg RNA, iScript Select cDNA Synthesis Kit and random oligonucleotide primers (Bio-Rad, United States). qRT-PCR was performed with cDNA, SsoAdvanced Universal SYBR Green Supermix (Bio-Rad, United States) and target-specific primers (Supplementary Table S2) in CF96 Real-Time System as following: 1 cycle of 95°C for 5 min, 40 cycles of 95°C for 10 s, 45°C for 20 s and 70°C for 30 s. All expression data were normalized to the copy number of 16S rRNA in each sample (Wen et al., 2021).

### Determining antifungal activity

Strains were cultured in LB medium at 37°C and 180 rpm for 48 h. After centrifugation at 6,500 g for 10 min, the supernatant of broths were collected for determining the antifungal activity against *F. oxysporum*. Briefly, PDA plates containing the broth supernatant at a ratio of 10% (v/v) were used for culturing *F. oxysporum* at 28°C for 4 days, then the diameter of colony was determined. PDA plates without the broth supernatant were inoculated with *F. oxysporum* as control. The inhibition rate of broth supernatant was calculated by the following formula: Inhibition rate = (Colony diameter in control−Colony diameter in broth supernatant)/Colony diameter in control × 100%.

### Fermentation optimization

Carbon sources, nitrogen sources, amino acids and inorganic salts in the medium were optimized for increasing antifungal activity and antifungal lipopeptides production, respectively. On the basis of medium optimization, we further studied the effect of culture conditions including temperature, initial pH, amounts of inoculation and ventilation on the antifungal activity and lipopeptides production, respectively. The detailed methods were described in Supplementary Materials.

### Fermentation in bioreactor

The fermentation was batch culture with 30 L optimized medium containing 300 ml defoamer (Soybean oil) in a 50 L fermenter (GJBioTech company, Shanghai, China). Briefly, the engineered strain Δ*kinA*Δ*bdh*Δ*dhbF*Δ*rapA*/T2-*sfp* was cultured in LB medium at 37°C and 180 rpm overnight, then the broth was transferred into 400 ml fresh LB medium in 1 L—flask at a ratio of 1% (v/v). After culturing for 8 h, the fresh broth was inoculated into optimized medium at a ratio of 2% (v/v). The fermentation was performed at 37°C with an agitation speed of 180 rpm and an aeration rate of 0.5 vvm for 48 h. In this period, the broth was collected for detecting the antifungal activity, pH and biomass (OD_600_ value), respectively.

### Purification and identification of antifungal lipopeptides

Antifungal lipopeptides were purified from the broth fermented in 50 L—bioreactor as described above. Briefly, 200 ml broth supernatant was adjusted to pH 2.0 with 6 M HCl for precipitating lipopeptides, then the precipitates were dissolved in 20 ml pure water and extracted by the same volume of *n*-butanol. The extracted substances were loaded into silica gel (FCP-200) column then eluted by *n*-butanol: ethanol: acetic acid: water = 30:70:5:20 (v/v) (Xing et al., 2018). The eluted fractions with iturin and fengycin were monitored by measuring the absorbance at 210 nm wavelength and the antifungal activity against *F. oxysporum*. Briefly, the fractions were dried by vacuum rotary evaporation, then the residual powers were dissolved in 2 ml pure water. After filter sterilization, 10 μl solution was loaded onto the filter paper disk with a diameter of 5 mm, then plated on the PDA plates inoculated with *F. oxysporum* to determining the antifungal activity*.*

The fractions with antifungal activity were further separated by HPLC (LC-100, WUFENG instruments, Shanghai, China) plus a Venusil MP C_18_ column (10 × 250 mm, 5 μm) using the mobile phase acetonitrile : 0.1% trifluoroacetic acid = 40 : 60 (v/v). The eluted fractions with iturin and fengycin were monitored by the antifungal activity against *F. oxysporum* as described above. Finally, the purified lipopeptides were identified by MALDI-TOF-MS (Li et al., 2006; Li et al., 2022).

### Quantitative analysis of antifungal lipopeptides by HPLC

10 ml broth of WH1 and the engineered strain Δ*kinA*Δ*bdh*Δ*dhbF*Δ*rapA*/T2-*sfp* cultured in flask or bioreactor were centrifuged for collecting supernatants, then the pH of supernatants were adjusted to pH 2.0 for precipitating lipopeptides as above. The precipitates were dissolved in 10 ml methanol, then the supernatants were collected by centrifugation at 8,000 g for 5 min. The content of iturin and fengycin in supernatants was analyzed by HPLC (Shimadzu, Japan) equipped with a Venusil MP C_18_ column (4.6 × 250 mm, 5 μm) using the mobile phase acetonitrile: 0.1% trifluoroacetic acid = 40:60 (v/v). The purified iturin and fengycin described above were used as standards for counting the content of lipopeptides in samples.

### Statistical analysis of data

All experiments are repeated in triplicates. Data between two groups were compared by a student *t*-test with a significant level of *p < 0.05 and **p < 0.01. Differences in multiple groups were analyzed by Analysis of Variance (ANOVA), and different letters indicate significant differences among different groups.

## Results

### Iturin and fengycin contributed most of antifungal activity to B. amyloliquefaciens

On the basis of ∆*ituB* and ∆*fenA*, we constructed the double knockout strain ∆*ituB*∆*fenA*, then the antifungal activity was detected for these strains. It was found that both ∆*ituB* and ∆*fenA* showed a slightly weaker antifungal activity than the wild-type strain WH1, while the double knockout strain ∆*ituB*∆*fenA* almost lost the antifungal activity against *F. oxysporum* (Supplementary Figure S2). These results clearly showed that iturin and fengycin contribute most of the antifungal activity to *B. amyloliquefaciens* WH1. Thereby, we used the antifungal activity to asses the antifungal lipopeptides (iturin and fengycin) production in this study.

### Increasing antifungal activity by enhancement of branched-chain amino acids biosynthesis

**FIGURE 2:**
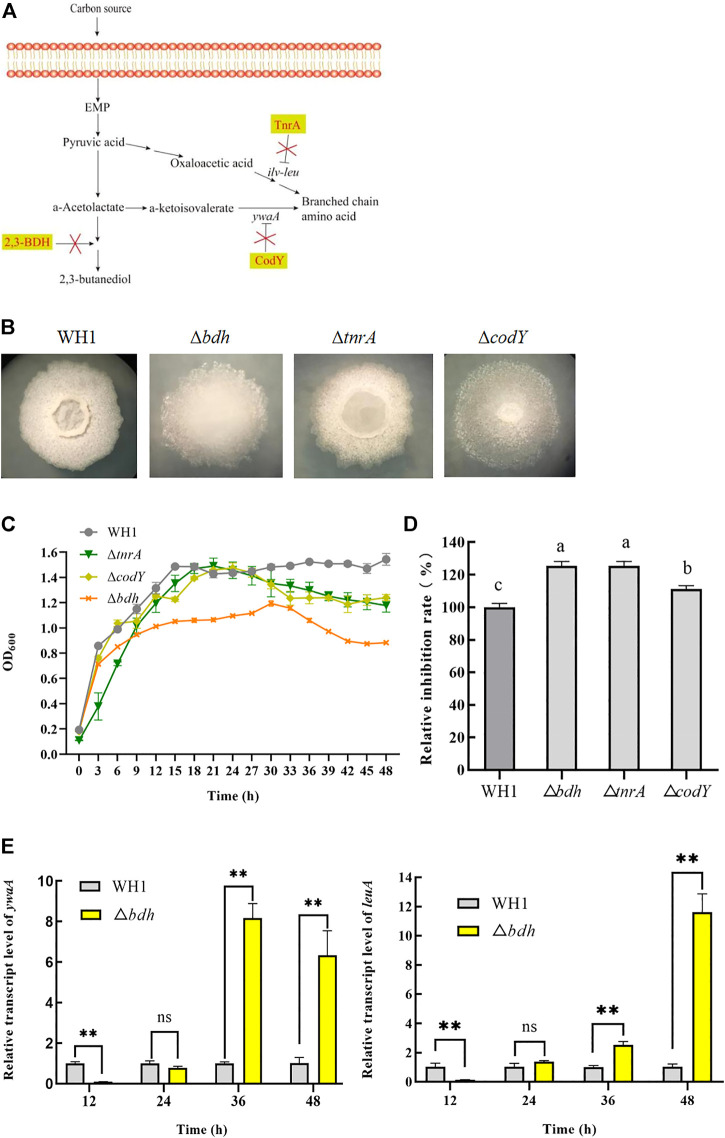
Relative inhibition rates and characteristic of knockout strains related with biosynthesis of branched-chain amino acids. **(A)**: Metabolic pathway and regulation of carbon overflow metabolism and biosynthesis of branched-chain amino acids. 2,3-BDH: 2,3-butanediol dehydrogenase; TnrA and CodY: regulators for carbon and nitrogen metabolism; *ywaA:* the aminotransferase gene; *ilv*-*leu*: the operon for biosynthesis of branched-chain amino acids. **(B)**: Colony morphology. **(C)**: Growth curves. **(D)**: Relative inhibition rates. **(E)**: Relative transcription level of *ywaA* and *leuA* in ∆*bdh.*

Branched-chain amino acids are crucial component of lipopeptides such as surfactin, iturin and fengycin. Here, we deleted *bdh*, *tnrA* and *codY*, respectively, to increase the supply of branched-chain amino acids (Figures 2A,B). The result showed that deletion of *bdh* encoding 2,3-butanediol dehydrogenase led to a weaker growth than WH1, while knockout of *codY* (encoding a regulator CodY for carbon and nitrogen metabolism) or *tnrA* (encoding a regulator TnrA for nitrogen metabolism) had no significant influence on the cell growth (Figure 2C).

Deletion of *bdh* resulted in an increase of antifungal activity (Figure 2D). Compared to that of WH1, the antifungal activity of ∆*bdh* was increased by 24%. The transcription of *ywaA* and *leuA,* two genes encoding key enzymes for biosynthesis of branched-chain amino acids, were both significantly up-regulated in ∆*bdh* at 36 and 48 h when compared to that of WH1 (Figure 2E). CodY and TnrA inhibit the biosynthesis of branched-chain amino acids. Consistently, deletion of *codY* and *tnrA*, respectively, resulted in an increase of antifungal activity in this study (Figure 2D). Compared to that of WH1, the antifungal activity of ∆*tnrA* was increased by 24%.

### Disruption of sporulation enhanced biosynthesis of antifungal lipopeptides

**FIGURE 3:**
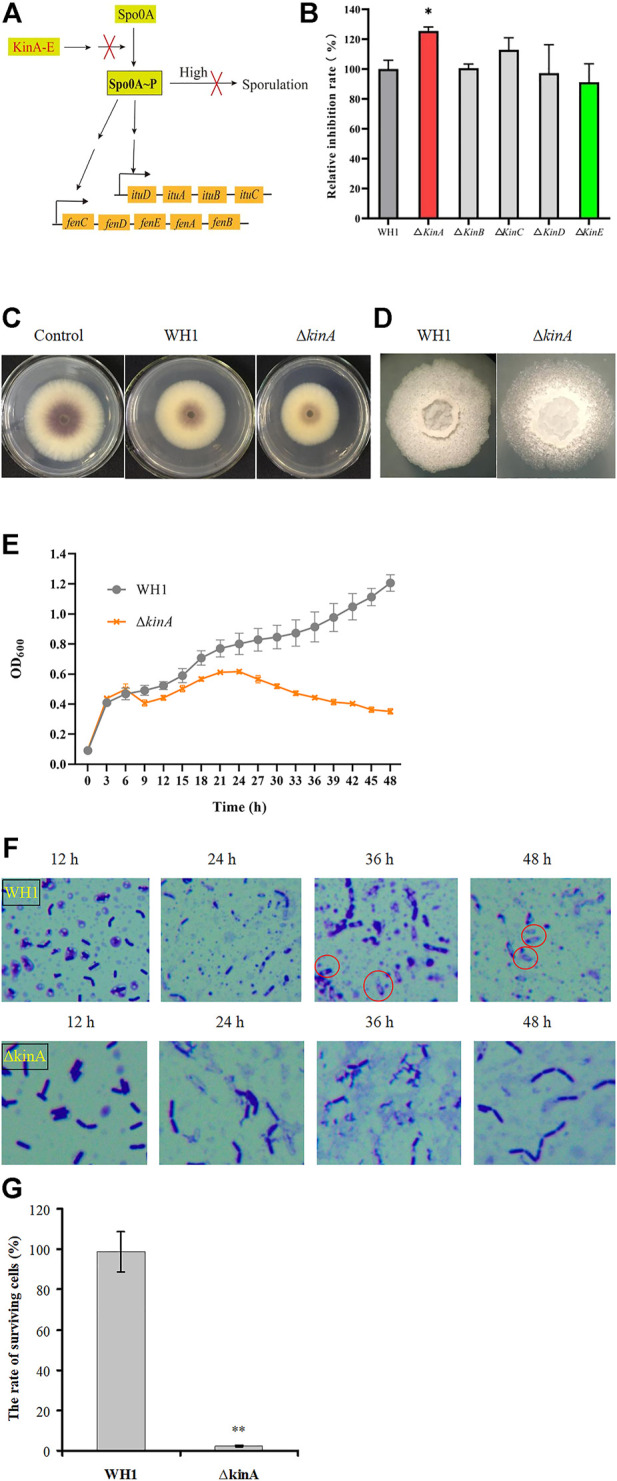
Relative inhibition rates and characteristics of the *kinA* knockout strain. **(A)**: Activation of Spo0A by Kin A-E. Arrows mean the promotion role; T-bars men the inhibition role; “High” means the high level of Spo0A∼P. **(B)**: Relative inhibition rates. **(C)**: Antifungal activity determined on PDA plates. **(D)**: Colony morphology. **(E)**: Growth curves. **(F)**: Sporulation. **(G)**: The rate of surviving cells after heating.

Once sporulation, the biosynthesis of secondary metabolites will be remarkably reduced. In order to extend the stage for producing secondary metabolites, we deleted 5 kinase genes (*kin A* to *E*) involved in activation of Spo0A, a global regulator that is essential for triggering sporulation (Figure 3A). Deletion of *kinB*, *C*, *D* and *E* had no significant influence on the antifungal activity, respectively, but knockout of *kinA* resulted in a significant increase of antifungal activity (Figure 3B). Compared to that of WH1, the antifungal activity of ∆*kinA* was increased by 10% (Figure 3C). The colony morphology of ∆*kinA* was slightly different from WH1 (Figure 3D), but the growth of ∆*kinA* was obviously weaker than WH1 (Figure 3E). KinA is a main histidine kinase for sporulation. Consistently, deletion of *kinA* blocked the spore generation in WH1 (Figure 3F). After heating at 80 ^o^C for 10 min, the rate of surviving cells was 98.78% for WH1, while it was only 2.33% for ∆*kinA* (Figure 3G), further confirmed that deletion of *kinA* disrupted the sporulation in WH1.

### Double knockout of kinA and bdh further enhanced antifungal activity

**FIGURE 4:**
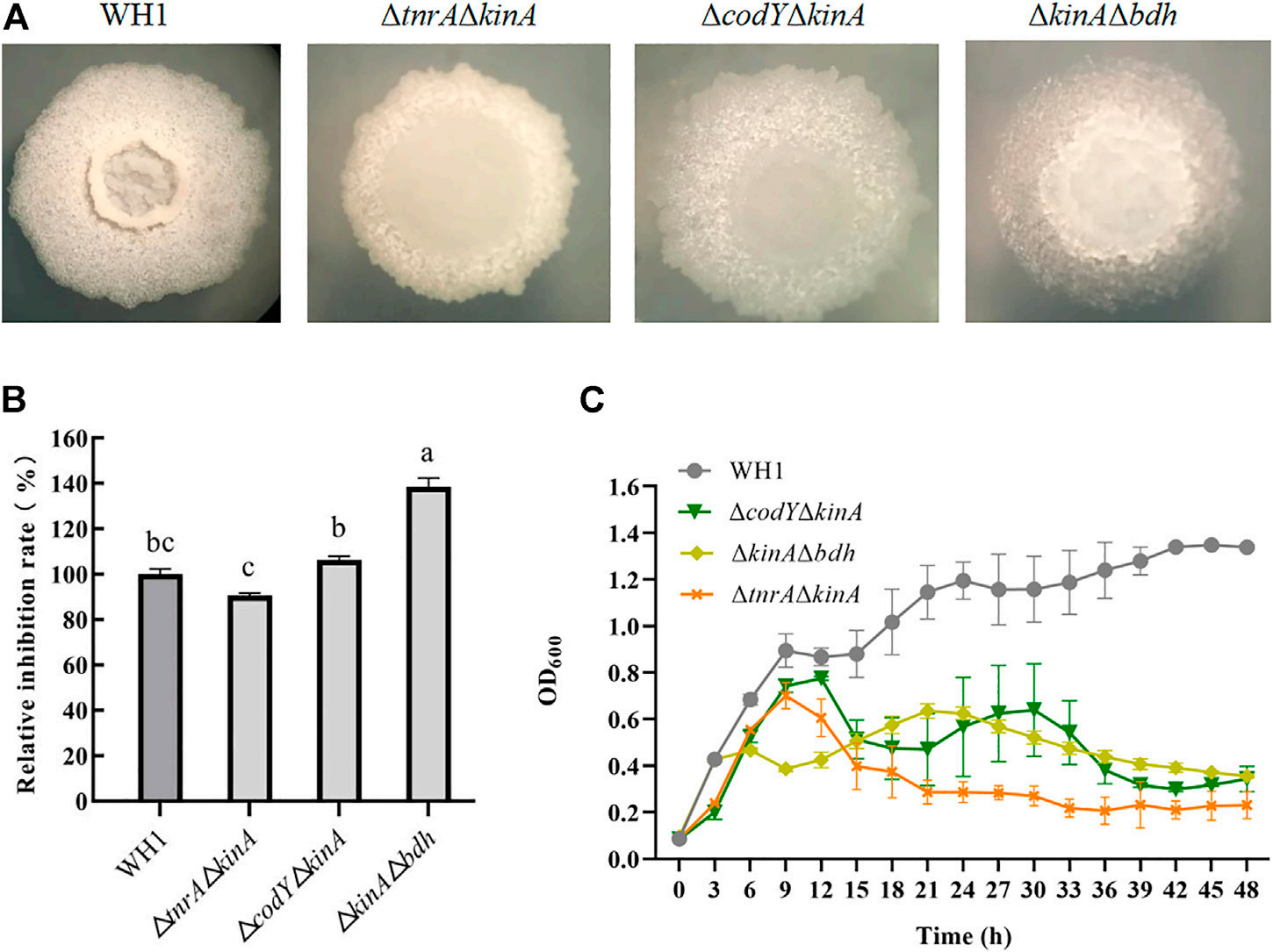
Relative inhibition rates and characteristics of double-knock strains. **(A)**: Colony morphology; **(B)**: Relative inhibition rates; **(C)**: Growth curves.

Deletion of *bdh*, *tnrA*, *codY* and *kinA*, respectively, could increase the antifungal activity. We further constructed the double knockout stains including Δ*tnrA*Δ*kinA*, Δ*codY*ΔkinA and Δ*kinA*Δ*bdh* here (Figure 4A). The antifungal activity of Δ*tnrA*Δ*kinA* was lower than WH1, while Δ*codY*Δ*kinA* had a similar antifungal activity to WH1. However, Δ*kinA*Δ*bdh* showed a significantly higher antifungal activity than WH1 (Figure 4B). Compared to that of WH1, the antifungal activity of Δ*kinA*Δ*bdh* was increased by 38%. Due to deletion of *kinA*, the growth of double-knockout strains were all weaker than WH1 (Figure 4C).

### Disruption of biofilm formation strengthened antifungal activity

**FIGURE 5:**
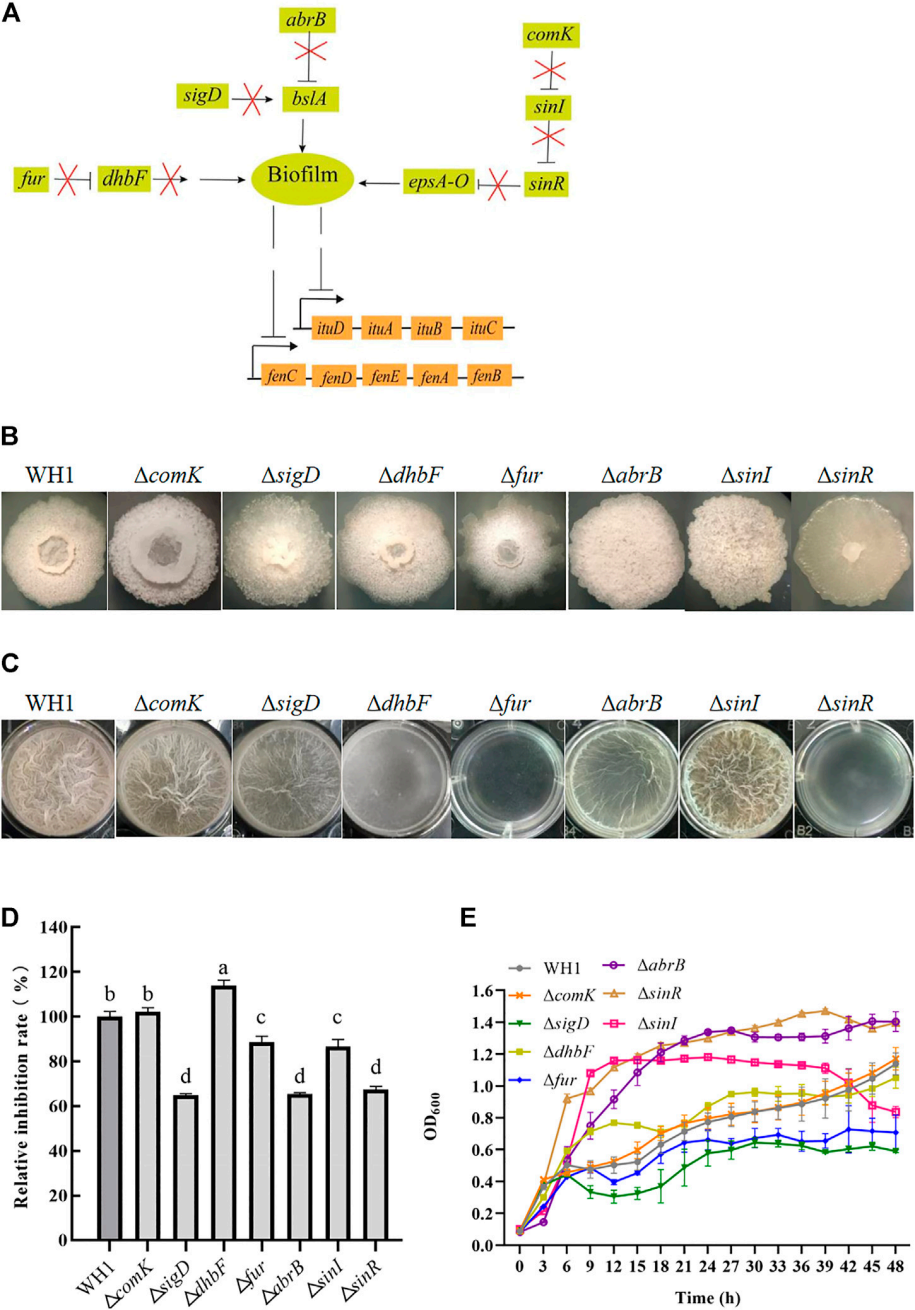
Relative inhibition rates and characteristics of knockout strains related with biofilm formation. **(A)**: Regulation of biofilm formation. The *bslA* gene encodes an extracellular protein of biofilm; the *epsA*-*O* operon encodes the enzymes for biosynthesizing exopolysaccharides of biofilm. ComK: competence transcription factor; SigD: sigma factor D; DhbF: enzyme for biosynthesis of siderophore; Fur: repressor of *dhb*; AbrB and SinR: regulators to suppress biofilm formation; SinI: regulator to promote biofilm formation. **(B)**: Colony morphology. **(C)**: Biofilms. **(D)**: Relative inhibition rates. **(E)**: Growth curves.

As illustrated in Figure 5A, we knocked out several genes related to biofilm formation such as *comK*, *sigD*, *dhbF* and *fur*, *abrB* and *sinR*, and *sinI*. Deletion of respective genes resulted in an obvious change of colony morphology (Figure 5B) and float pellicle (Figure 5C). Deletion of *dhbF*, *fur*, *abrB* and *sinR*, respectively, resulted in an obvious decrease of biofilm compared to that of WH1, while knockout of *sinI* led to a robuster float pellicle than WH1 (Figure 5C). Further analysis showed that only the antifungal activity of Δ*dhbF* (*dhbF* belonging to the *dhb* gene cluster for biosynthesis of siderophore) was increased by 13% compared to that of WH1 (Figure 5D). The growth curves showed deletion of *dhbF* had no significant influence on the cell growth (Figure 5E). Deletion of *sinI* could not improve the antifungal activity in WH1.

### Triple knockout strain ΔkinAΔbdhΔdhbF with strengthened antifungal activity

**FIGURE 6:**
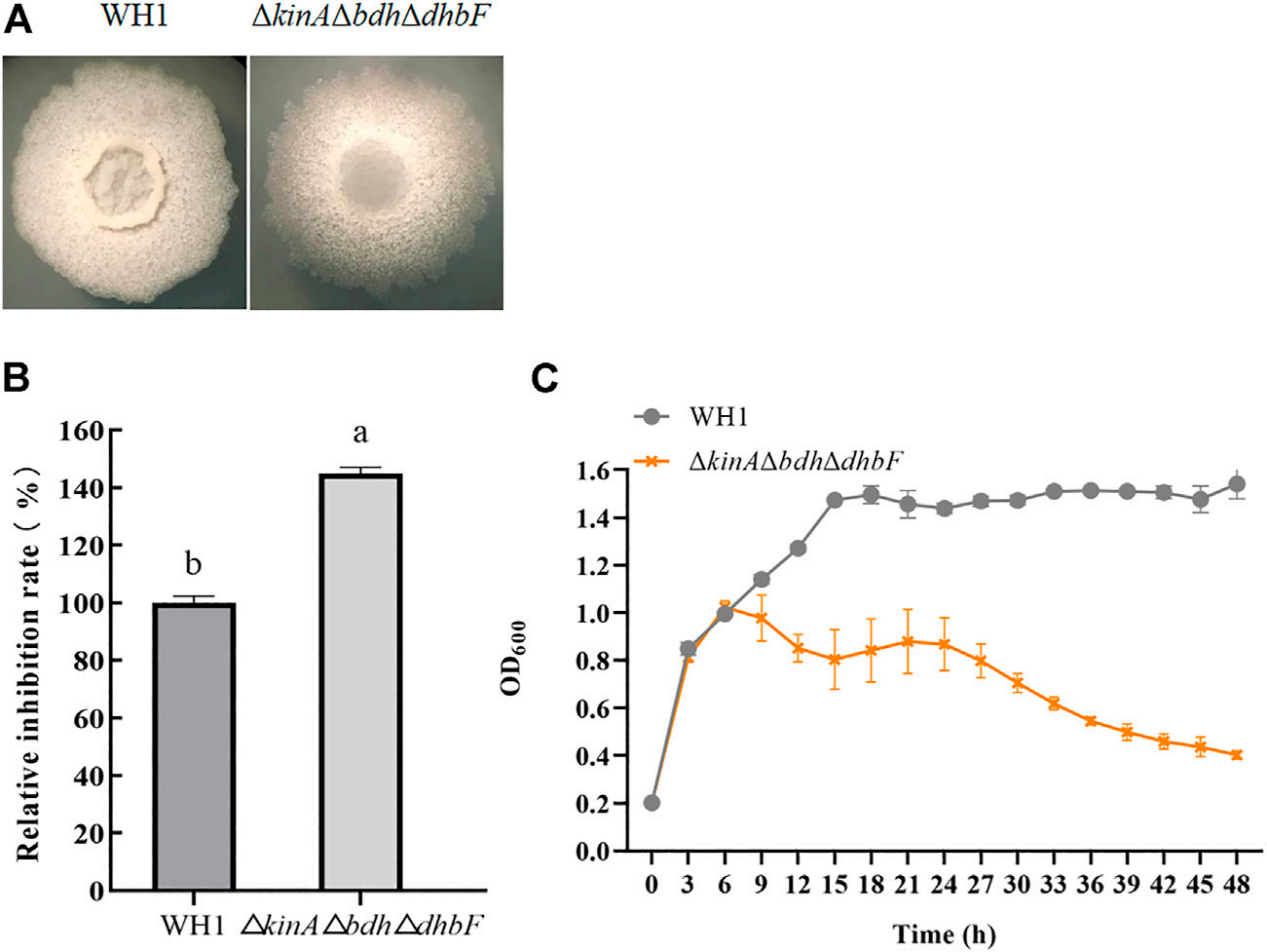
Relative inhibition rate and characteristic of Δ*kinA*Δ*bdh*Δ*dhbF*. **(A)**: Colony morphology; **(B)**: Relative inhibition rate; **(C)**: Growth curve.

For further improving the antifungal activity, we constructed the triple knockout stain Δ*kinA*Δ*bdh*Δ*dhbF* on the basis of Δ*kinA*Δ*bdh* (Figure 6A). Δ*kinA*Δ*bdh*Δ*dhbF* had a significantly higher antifungal activity than WH1 (Figure 6B). Compared to that of WH1, the antifungal activity was increased by 44% in Δ*kinA*Δ*bdh*Δ*dhbF*. Due to deletion of *kinA*, Δ*kinA*Δ*bdh*Δ*dhbF* also showed a weaker growth than WH1 (Figure 6C).

### Elevation of Spo0A∼P improved antifungal activity

**FIGURE 7:**
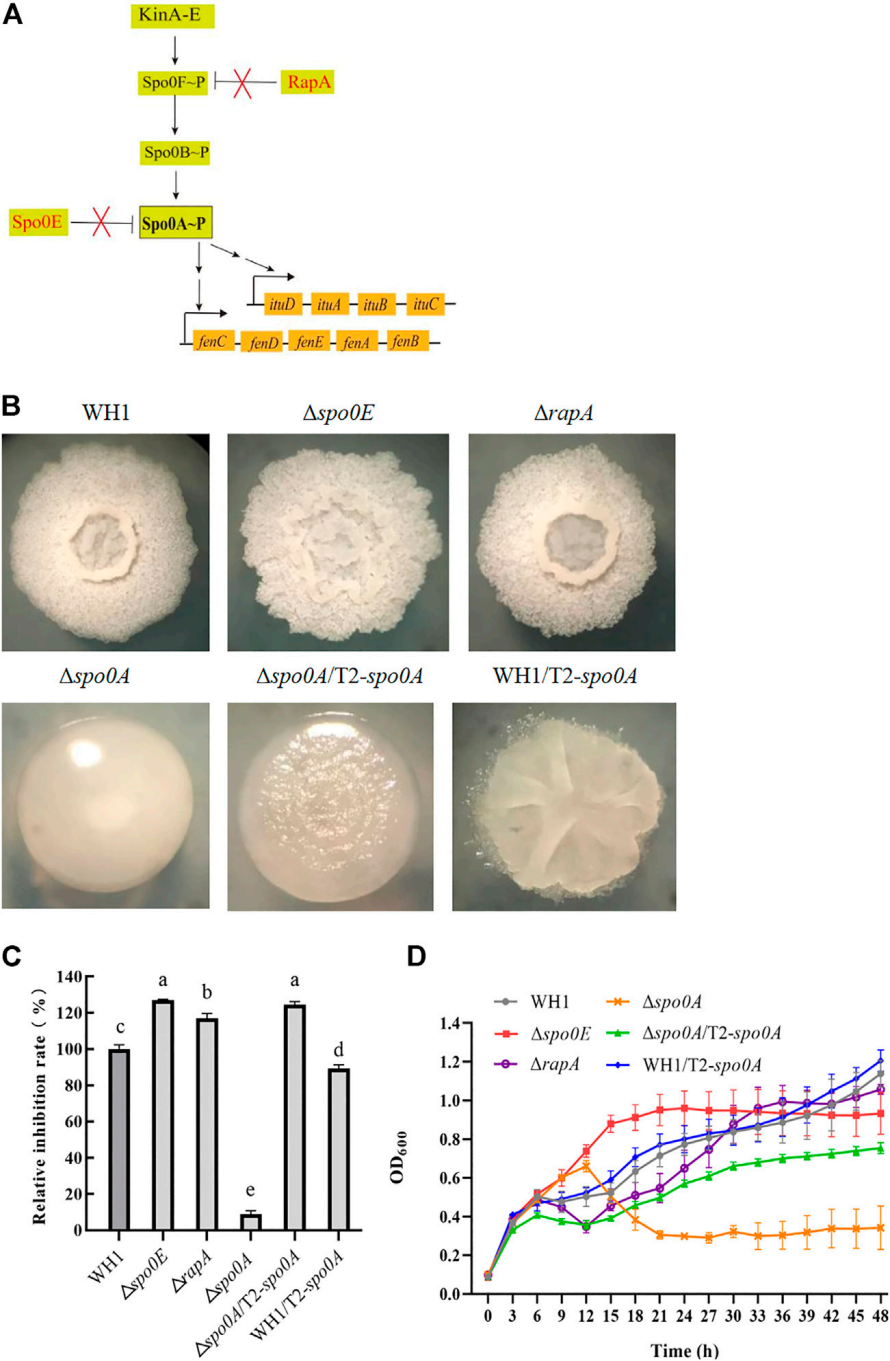
Relative inhibition rates and characteristics of engineered strains related with Spo0A phosphorylation. **(A)**: Phosphorylation and dephosphorylation of Spo0A. Spo0F and Spo0B are the phosphate group transporters. **(B)**: Colony morphology. **(C)**: Relative inhibition rates. **(D)**: Growth curves.

Spo0A∼P regulate biosynthesis of many secondary metabolites. Here, we deleted *spo0A* in WH1 (Figure 7B), and found the ability to produce antifungal lipopeptides was significantly reduced in Δ*spo0A* compared to WH1 (Figure 7C). As illustrated in Figure 7A, the aspartate phosphatase family RapA can indirectly dephosphorylate Spo0A∼P by dephosphorylating the phosphate group transporter Spo0F∼P, and the phosphatase Spo0E is able to directly dephosphorylate Spo0A∼P to Spo0A. Here, both deletion of *spo0E* and *rapA* (Figure 7B) could significantly increase the antifungal activity in WH1 (Figure 7C). Except for Δ*spo0A*, the growth of Δ*spo0E* and Δ*rapA* were both similar to WH1 (Figure 7D).

Due to the importance of Spo0A for antifungal activity, we overexpressed *spo0A* in Δ*spo0A* and WH1, respectively. The results showed that compensation with *spo0A* could not restore the colony morphology (Figure 7B), but was able to significantly increase the antifungal activity of Δ*spo0A* (Figure 7C). Also, the impaired growth of Δ*spo0A* was well restored by compensation with *spo0A* (Figure 7D). However, overexpression of *spo0A* led to a markedly different colony morphology from WH1 (Figure 7B). Moreover, overexpression of *spo0A* resulted in a decrease of antifungal activity (Figure 7C), but had no significant influence on the cell growth (Figure 7D). Accordingly, overexpression of *spo0A* for increasing lipopeptides production should be done in Δ*spo0A* rather than in WH1.

### Further enhanced antifungal activity in ΔkinAΔbdhΔdhbFΔrapA

**FIGURE 8:**
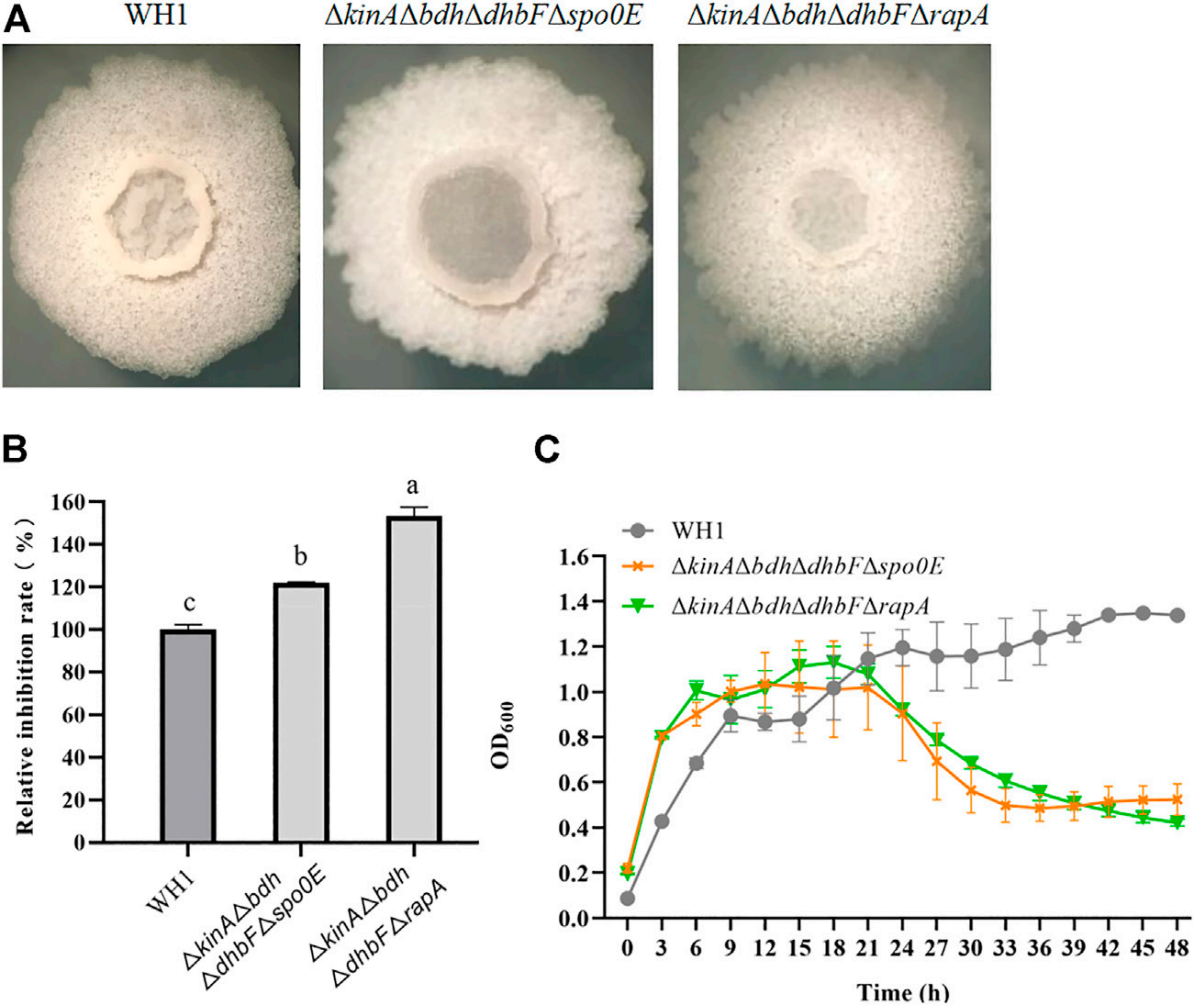
Relative inhibition rates and characteristics of quadruple knockout strains. **(A)**: Colony morphology; **(B)**: Relative inhibition rates; **(C)**: Growth curves.

On the basis of Δ*kinA*Δ*bdh*Δ*dhbF*, we constructed the quadruple knockout strains Δ*kinA*Δ*bdh*Δ*dhbF*Δ*spo0E* and Δ*kinA*Δ*bdh*Δ*dhbF*Δ*rapA,* respectively (Figure 8A). Deletion of *spo0E* in Δ*kinA*Δ*bdh*Δ*dhbF* could not further increase the antifungal activity. Conversely, it led to a decrease of antifungal activity. However, deletion of *rapA* in Δ*kinA*Δ*bdh*Δ*dhbF* could further improve the antifungal activity (Figure 8B). Compared to that of WH1, the antifungal activity was increased by 53% in Δ*kinA*Δ*bdh*Δ*dhbF*Δ*rapA*. The growth of Δ*kinA*Δ*bdh*Δ*dhbF*Δ*spo0E* and Δ*kinA*Δ*bdh*Δ*dhbF*Δ*rapA* were both better than Δ*kinA*, Δ*kinA*Δ*bdh* and Δ*kinA*Δ*bdh*Δ*dhbF* (Figure 8C).

### Overexpression of sfp increased antifungal activity

**FIGURE 9:**
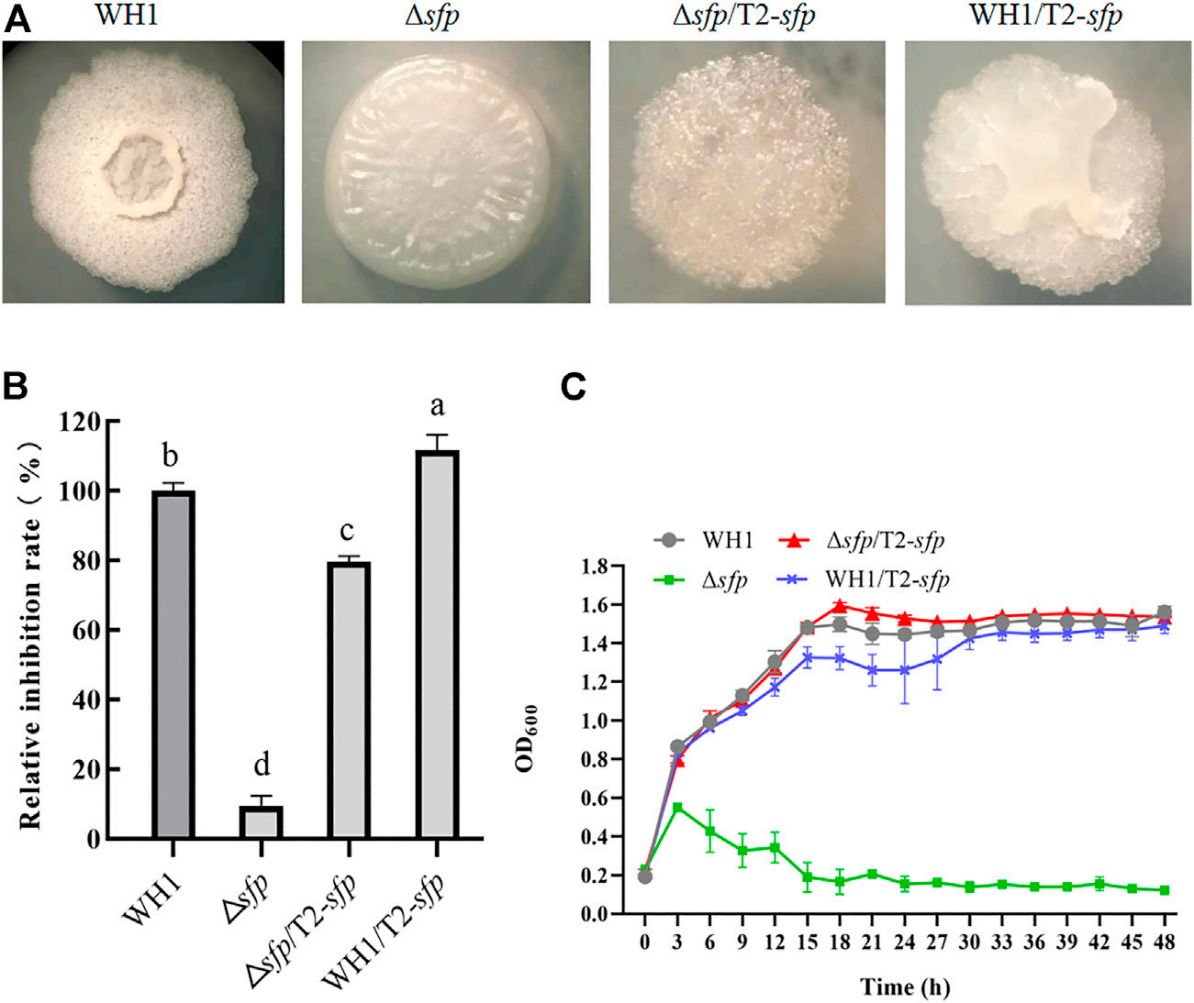
Relative inhibition rates and characteristics of engineered strains related with *sfp*. **(A)**: Colony morphology; **(B)**: Relative inhibition rates; **(C)**: Growth curves.

4-Phosphopantetheinyl transferase (Sfp) is essential for biosynthesis of lipopeptides. Here, deletion of *sfp* led to an obvious change of colony morphology in WH1 (Figure 9A). After deletion of *sfp*, the antifungal activity was also significantly decreased in WH1 (Figure 9B). Moreover, the growth of Δ*sfp* was impaired compared to that of WH1 (Figure 9C). Thereby, Sfp is essential for biosynthesis of antifungal lipopeptides and showing antifungal activity in *B. amyloliquefaciens*.

For further improving the antifungal activity, we overexpressed *sfp* in Δ*sfp* and WH1, respectively. Interestingly, compensation with *sfp* could not restore the colony morphology of Δ*sfp*, and overexpression of *sfp* led to a changed colony morphology in WH1 (Figure 9A). Moreover, compensation with *sfp* only restored the antifungal activity in Δ*sfp*, while overexpression of *sfp* could further improve the antifungal activity in WH1 (Figure 9B). Compared to that of WH1, the antifungal activity was increased by 12% in the overexpression strain WH1/T2-*sfp*. Compensation with *sfp* could restore the growth of Δ*sfp* (Figure 9C).

### Antifungal activity was further strengthened in ΔkinAΔbdhΔdhbFΔrapA/T2-sfp

**FIGURE 10:**
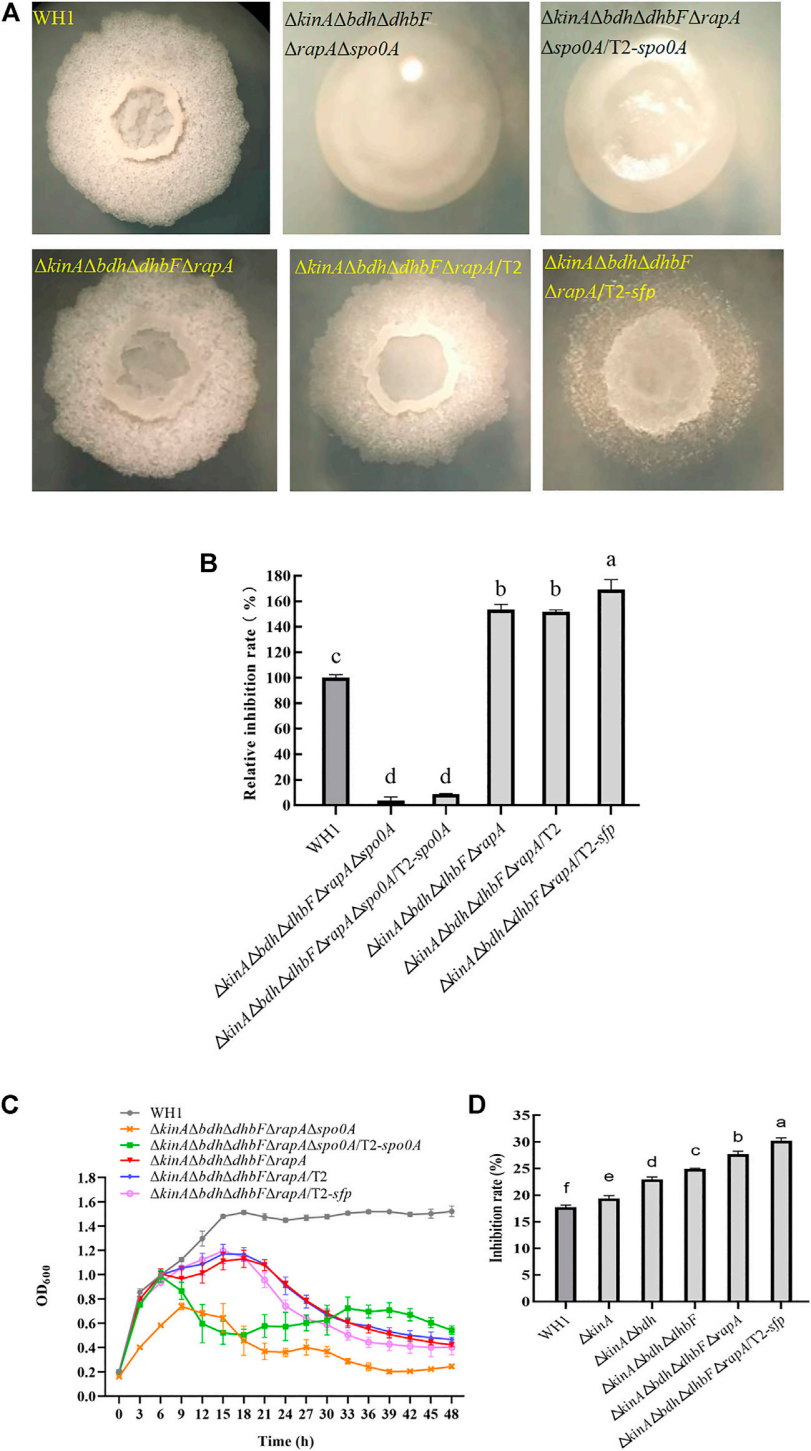
Relative inhibition rates and characteristics of Δ*kinA*Δ*bdh*Δ*dhbF*Δ*rapA*/T2-*sfp*. **(A)**: Colony morphology; **(B)**: Relative inhibition rates; **(C)**: Growth curves; **(D)**: Comparison of antifungal activity among different engineered strains (from the original strain WH1 to the final strain Δ*kinA*Δ*bdh*Δ*dhbF*Δ*rapA*/T2-*sfp*).

We further overexpressed *spo0A* and *sfp* in Δ*kinA*Δ*bdh*Δ*dhbF*Δ*rapA*Δ*spo0A* and Δ*kinA*Δ*bdh*Δ*dhbF*Δ*rapA*, respectively. First, we constructed the penta knockout strain Δ*kinA*Δ*bdh*Δ*dhbF*Δ*rapA*Δ*spo0A* on the basis of Δ*kinA*Δ*bdh*Δ*dhbF*Δ*rapA* (Figure 10A). Δ*kinA*Δ*bdh*Δ*dhbF *Δ*rapA*Δ*spo0A* showed a significant decease of antifungal activity due to deletion of *spo0A* (Figure 10B). Second, we transferred T2 plasmid into Δ*kinA*Δ*bdh*Δ*dhbF*Δ*rapA* as a control to rule out the possible influence induced by this plasmid (Figure 10A). The result showed the antifungal activity of Δ*kinA*Δ*bdh*Δ*dhbF*Δ*rapA*/T2 had no significant difference from the host Δ*kinA*Δ*bdh*Δ*dhbF*Δ*rapA* (Figure 10B).

Δ*kinA*Δ*bdh*Δ*dhbF*Δ*rapA*Δ*spo0A*/T2-*spo0A* and Δ*kinA*Δ*bdh*Δ *dhbF*Δ*rapA*/T2-*sfp* was constructed on the basis of Δ*kinA*Δ*bdh*Δ*dhbF*Δ*rapA*Δ*spo0A* and Δ*kinA*Δ*bdh*Δ*dhbF*Δ*rapA,* respectively (Figure 10A). Δ*kinA*Δ*bdh*Δ*dhbF*Δ*rapA*Δ*spo0A*/T2-*spo0A* had a similar antifungal activity to Δ*kinA*Δ*bdh*Δ*dhbF*Δ*rapA*Δ*spo0A*, indicating that compensation with *spo0A* could not restore the antifungal activity in this strain (Figure 10B). However, compensation with *spo0A* could partially restore the growth of Δ*kinA*Δ*bdh*Δ*dhbF*Δ*rapA*Δ*spo0A* (Figure 10C).

Δ*kinA*Δ*bdh*Δ*dhbF*Δ*rapA*/T2-*sfp* showed a significantly higher antifungal activity than Δ*kinA*Δ*bdh*Δ*dhbF*Δ*rapA*, indicating that overexpression of *sfp* could further improve the antifungal lipopeptides production in Δ*kinA*Δ*bdh*Δ*dhbF*Δ*rapA*. Compared to that of WH1, the antifungal activity was increased by 65% in Δ*kinA*Δ*bdh*Δ*dhbF*Δ*rapA*/T2-*sfp* (Figure 10B). However, its growth was similar to Δ*kinA*Δ*bdh*Δ*dhbF*Δ*rapA*, which were both weaker than WH1 (Figure 10C).

We further verified the antifungal activity of engineered strains, including Δ*kinA*, Δ*kinA*Δ*bdh*, Δ*kinA*Δ*bdh*Δ*dhbF*, Δ*kinA*Δ*bdh*Δ*dhbF*Δ*rapA* and Δ*kinA*Δ*bdh*Δ*dhbF*Δ*rapA*/T2-*sfp*. From WH1 to Δ*kinA*Δ*bdh*Δ*dhbF*Δ*rapA*/T2-*sfp*, the antifungal activity was increased step by step. The antifungal activity was improved by 1.7-fold in the final strain Δ*kinA*Δ*bdh*Δ*dhbF*Δ*rapA*/T2-*sfp* compared to that in the original strain WH1 (Figure 10D).

### Fermentation optimization significantly enhanced antifungal activity

We selected the modified Landy as an initial medium for Δ*kinA*Δ*bdh*Δ*dhbF*Δ*rapA*/T2-*sfp* to produce antifungal lipopeptides. After optimization, the final medium formula for culturing Δ*kinA*Δ*bdh*Δ*dhbF*Δ*rapA*/T2-*sfp* to produce antifungal lipopeptides contains 20 g/L glucose, 20 g/L soybean meal power, 0.5 g/L MgSO_4_•7H_2_O, 1.0 g/L KH_2_PO_4_, 0.8 g/L ZnSO_4_•7H_2_O and 7.0 mg/L MnSO_4_•H_2_O in 1 L water, pH 8.0. In this formula, the inhibition rate of broth achieved at 37.18%. On the basis of above medium, the fermentation conditions were also optimized, including temperature (37°C), initial pH value (8.0), inoculation rate (2%), liquid volume (100 ml medium loaded in 250 ml flask) and fermentation time (48 h). The detailed results were described in the Supplementary Materials.

### Antifungal lipopeptides produced by ΔkinAΔbdhΔdhbFΔrapA/T2-sfp

**FIGURE 11:**
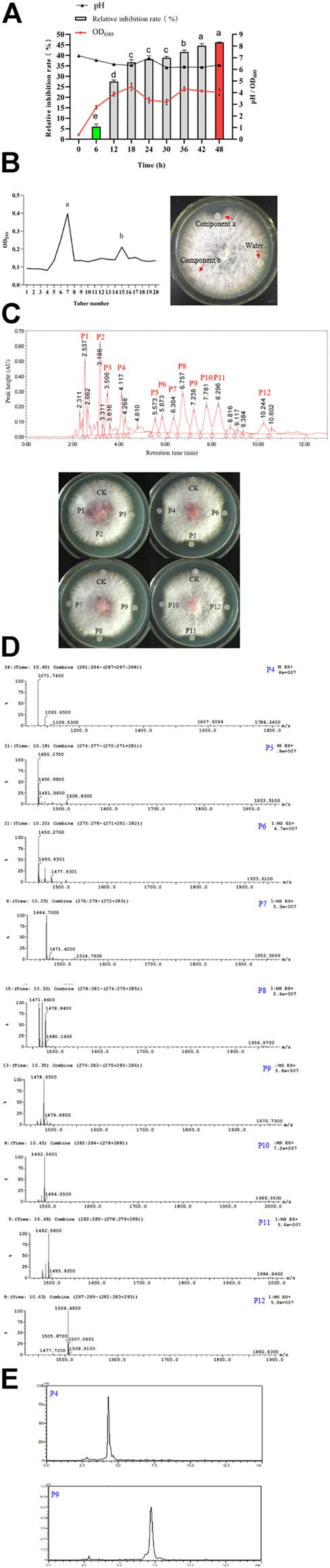
Fermentation, purification and identification of antifungal lipopeptides. (**A)**: Fermentation of antifungal lipopeptides in bioreactor. **(B)**: Separation of antifungal lipopeptides by silica gel column. Left: The extracted substances in *n*-butanol were separated by silica gel column; Right: Antifungal activity of crude lipopeptides separated by silica column. **(C)**: Purifying antifungal lipopeptides by RP-HPLC. Top: P1–P12 were the peaks with a retention time 2.537, 3.186, 3.506, 4.117, 5.573, 5.873, 6.364, 6.757, 7.238, 7.781, 8.296 and 10.244 min, respectively; Bottom: Antifungal activity of P1–P12. CK: control (water). **(D)**: Analysis of P4–P12 by mass spectrometry. **(E)**: Determination of the purity of iturin (P4) and fengycin (P9) by HPLC.

We further verified the optimized fermentation medium and conditions in a 50 L- bioreactor. Δ*kinA*Δ*bdh*Δ*dhbF*Δ*rapA*/T2-*sfp* was cultured at 37°C with an agitation speed of 180 rpm and an aeration rate of 0.5 vvm. The results showed the biomass achieved at the maximum at 18 h, and the antifungal activity of broth was significantly increased from 12 h and reached the maximum at 48 h. The pH of broth was slightly decreased during the period of fermentation (Figure 11A).

The broth was used for extracting the antifungal lipopeptides by *n*-butanol, then separated by silica column. After elution, two components, a and b, were collected, but only Component a showed an obvious antifungal activity (Figure 11B). Component a was further purified by HPLC with C_18_ column, and could be separated into 12 peaks. After being dried by vacuum rotary evaporation, the residual powers were dissolved in pure water to a concentration of 1 mg/L for determining antifungal activity. The results showed that P4–P12 all showed an antifungal activity against *F. oxysporum* (Figure 11C). The antifungal substances in P4–P12 were determined by LC-MS, and the results showed P4–P12 were iturin and fengycin homologues, respectively (Figure 11D). P4 was iturin A homologue with C16 fatty acid chain, and P5–P12 were fengycin A homologues with C15–C18 fatty acid chains, and fengycin B homologues with C14–C17 fatty acid chains, respectively (Table 2).

**TABLE 2:** Characterization of lipopeptides by m/z in P4–P12.

Peak	Mass peak (m/z)	Ion type	Lipopeptide
P4	1071.74	[M + H]^+^	C16 Iturin A
P5	1450.17	[M + H]^+^	C15 Fengycin A
P6	1450.27	[M + H]^+^	C15 Fengycin A
P7	1464.70	[M + H]^+^	C16 Fengycin A
			C14 Fengycin B
P8	1471.46	[M + Na]^+^	C15 Fengycin A
P9	1478.65	[M + H]^+^	C17 Fengycin A
			C15 Fengycin B
P10	1492.56	[M + H]^+^	C18 Fengycin A
			C16 Fengycin B
P11	1492.58	[M + H]^+^	C18 Fengycin A
			C16 Fengycin B
P12	1506.48	[M + H]^+^	C17 Fengycin B

The purity of iturin and fengycin was further analyzed by HPLC with C_18_ column. P4 and P9 were detected for their purity. It was found that both P4 (Iturin A) and P9 (Fengycin A) showed a single peak after separation by HPLC (Figure 11E), suggesting these two lipopeptides were both at a high purity. Consequently, P4 and P9 were used as standards for quantitative analysis of iturin and fengycin in the broth.

### Antifungal lipopeptides production was significantly increased in the engineered strain

**FIGURE 12:**
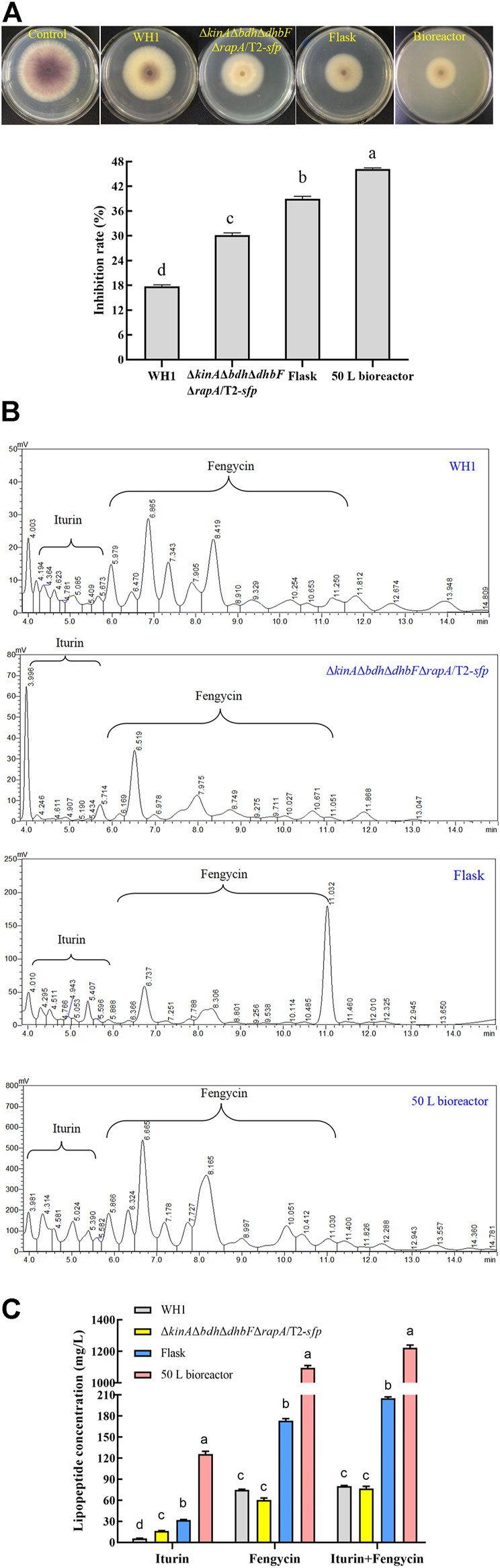
Comparison of antifungal activity and lipopeptides production between WH1 and Δ*kinA*Δ*bdh*Δ*dhbF*Δ*rapA*/T2-*sfp*. **(A)**: Antifungal activity. Top: Antifungal activity determined on PDA plates; Bottom: Inhibition rates. **(B)**: Analysis of lipopeptides in the broth by HPLC. **(C)**: Antifungal lipopeptides titer in the broth of WH1 and Δ*kinA*Δ*bdh*Δ*dhbF*Δ*rapA*/T2-*sfp*.

The purified iturin A (P4) and fengycin A (P9) were used as standards for making standard curves. The content of iturin and fengycin was quantitatively determined in the broth of WH1 and Δ*kinA*Δ*bdh*Δ*dhbF*Δ*rapA*/T2-*sfp,* respectively. The results showed the antifungal activity in Δ*kinA*Δ*bdh*Δ*dhbF*Δ*rapA*/T2-*sfp* was significantly higher than WH1. Also, the antifungal activity of Δ*kinA*Δ*bdh*Δ*dhbF*Δ*rapA*/T2-*sfp* in bioreactor was significantly higher that in flask (Figure 12A). By construction of the engineered strains and fermentation optimization, the antifungal activity was increased by 2.5-fold from 18% in WH1 to 46% in Δ*kinA*Δ*bdh*Δ*dhbF*Δ*rapA*/T2-*sfp*.

In the broth of WH1, the titer of iturin and fengycin was 5.4 mg/L and 75.2 mg/L, respectively, while the titer of iturin achieved at 17.0 mg/L in Δ*kinA*Δ*bdh*Δ*dhbF*Δ*rapA*/T2-*sfp* with an increase of 3.2-fold compared to that of WH1. After fermentation optimization in flask, the titer of iturin and fengycin in Δ*kinA*Δ*bdh*Δ*dhbF*Δ*rapA*/T2-*sfp* achieved at 31.1 mg/L and 175.3 mg/L, with an approximate increase of 1.8-fold and 2.3-fold, respectively. After fermentation in 50 L bioreactor, the titer of iturin and fengycin achieved at 123.5 mg/L and 1200.8 mg/L, which further improved by 4.0-fold and 6.8-fold, respectively (Figure 12B,C). Compared to that of WH1, the final iturin and fengycin titer of Δ*kinA*Δ*bdh*Δ*dhbF*Δ*rapA*/T2-*sfp* in bioreactor increased by 22.8-fold and 15.9-fold, respectively.

## Discussion

Many *Bacillus* species can produce antifungal lipopeptides such as iturin and fengycin against fungi (Kaspar et al., 2109). Previously, we isolated a strain WH1 with excellent antifungal activity, and was characterized as *B. amyloliquefaciens*. Here, we verified that the antifungal activity was mainly attributed to iturin and fengycin, consistent with the previous reports (Farzaneh et al., 2016; Liu et al., 2020). Therefore, we used antifungal activity to asses the antifungal lipopeptides (iturin and fengycin) production in this study.

Branched-chain amino acids are crucial component of lipopeptides. In *B. subtilis*, the biosynthesis of branched-chain amino acids is suppressed by CodY (a regulator to regulate both carbon metabolism and nitrogen metabolism) and TnrA (a regulator for nitrogen metabolism) (Fujita 2009; Fu et al., 2022). Knockout of *codY* can result in an increase of biosynthesis of branched-chain amino acids such as isoleucine, valine, etc (Brinsmade et al., 2014). Consistently, deletion of *codY* also led to an increase of antifungal activity in WH1. TnrA negatively regulates the expression of *ilv*-*leu* operon for biosynthesis of branched-chain amino acids (Tojo et al., 2005). Consistently, knockout of *tnrA* also led to a strengthening antifungal activity in WH1. For biosynthesis of lipopeptides, the pyruvate from glycolytic pathway is converted to the branched-chain amino acids, which are precursors for biosynthesis of several lipopeptides. However, pyruvate can also be sequentially converted to acetolactate, acetoin and 2,3-butanediol (Peng et al., 2019). Theoretically, deletion of the gene *bdh* encoding 2,3-butanediol dehydrogenase can increase the supply of pyruvate for biosynthesis of branched-chain amino acids. As expected, deletion of *bdh* resulted in an increase of antifungal activity here. It could be further verified by the result that the transcription of two genes *ywaA* and *leuA*, which encode key enzymes for biosynthesis of branched-chain amino acids, were both significantly up-regulated in Δ*bdh*. This might be explained by the reason that knockout of *bdh* could accumulate more pyruvate, which act as substrate to promote the expression of *ywaA* and *leuA* for biosynthesis of more branched-chain amino acids. Although Iturin A does not contain branched-chain amino acids, and fengycin contains only molecule isoleucine, surfactin contains several branched-chain amino acids. Generally, surfactin acts as a signal molecule for activation of Spo0A to form Spo0A∼P via histidine kinases (López et al., 2009). We found that maintaining Spo0A∼P level was favorable for increasing the antifungal lipopeptides production. Consequently, knockout of *bdh* could also increase the antifungal lipopeptides production here.

Once sporulation, the biosynthesis of secondary metabolites will be remarkably reduced in *Bacillus*. For example, the *spoIVB*-null non-spore-forming mutant of *B. subtilis* was especially efficient in producing the secondary metabolites such as surfactin (Wang et al., 2020). In order to extend the stage for producing secondary metabolites, we deleted 5 histidine kinase genes (*kin A* to *E*) involved in activation of Spo0A (a global regulator for triggering sporulation) to block sporulation, respectively (López et al., 2009). It was found that only knockout of *kinA* resulted in a significant increase of antifungal activity. In *B. subtilis*, KinA is a main histidine kinase for sporulation, and knockout of *kinA* can lead to a significant decrease of sporulation (Schultz, 2016). Consistently, deletion of *kinA* also blocked the spore generation in *B. amyloliquefaciens* WH1. We speculated that some cells were died due to the retardation for generating spores. As a result, the biomass was decreased in most of the strains with knockout of *kinA*. On the other hand, the disability to sporulate could extend the stage for producing lipopeptides.

The double knockout strains including *kinA* and one gene from *tnrA*, *codY* and *bdh* were constructed for further increasing antifungal activity. Three double knockout strains including Δ*tnrA*Δ*kinA*, Δ*codY*Δ*kinA* and Δ*bdh*Δ*kinA* were constructed, but only Δ*kinA*Δ*bdh* showed a significantly higher antifungal activity than WH1. The antifungal activity of Δ*tnrA*Δ*kinA* was lower than that of WH1, while Δ*codY*Δ*kinA* had a similar antifungal activity compared to WH1. KinA, TnrA and CodY are all global regulators, hence double knockout of *kinA* and *tnrA* or *codY* might cause negative influence on the cellular physiological and biochemical activities. However, deletion of *bdh* only blocked the carbon overflow metabolism to produce 2,3-butanediol, so double knockout of *kinA* and *bdh* had a weaker influence than other double knockout strains*.*

Biofilm is considered as a suppressor of lipopeptides (e.g., surfactin) synthesis, so disruption of biofilm formation is favorable for improving lipopeptides production (Wu et al., 2019). Here, we knocked out several genes related to biofilm formation such as *comK* (She et al., 2020), *sigD* (Fan et al., 2016), *dhbF* (a gene of *dhb* operon for biosynthesis of siderophore) and *fur* (a repressor of *dhb*) (Oliveira et al., 2017; Pi and Helmann 2017), *abrB* and *sinR* (regulators to suppress biofilm formation) (Newman et al., 2013; Klausmann et al., 2021), and *sinI* (a regulator to promote biofilm formation) (López et al., 2009). Deletion of respective genes resulted in an obvious change of colony morphology and float pellicle, but only deletion of *dhbF* led to a significant increase of antifungal activity. The results ruled out the possibility that reduction of biofilm formation could generally enhance antifungal activity. The positive effect of the *dhbF* null-mutation might be due to the blocking of siderophore synthesis, which enhances the availability of amino acids and fatty acids involved in lipopeptides synthesis (Oliveira et al., 2017; Pi and Helmann 2017). On this basis, the triple knockout stain Δ*kinA*Δ*bdh*Δ*dhbF* was constructed for further improving antifungal activity.

After phosphorylation, Spo0A regulates biosynthesis of many secondary metabolites (Rahman et al., 2006; Sun et al., 2021). For example, Klausmann et al. (2021) reported that the null-mutant of *spo0A* could reduce surfactin production in *B. subtilis*. Consistently, deletion of *spo0A* led to a very significant reduction of antifungal activity in WH1. This was different from knockout of *kinA*, which resulted in a significant increase of antifungal activity by disrupting the phosphorylation of Spo0A for sporulation. In *B. subtilis*, high levels of Spo0A∼P are essential for sporulation (López et al., 2009), so knockout of *kinA* only resulted in a decreased ability to phosphorylate Spo0A for sporulation, but Spo0A could still be phosphorylated via other pathways to produce lipopeptides. Due to the importance of Spo0A for antifungal activity, we overexpressed *spo0A* in Δ*spo0A* and WH1, respectively. Compensation with *spo0A* could significantly increase the antifungal activity in Δ*spo0A*, but overexpression of *spo0A* led to a decrease of antifungal activity in WH1. This result is different from the previous report that overexpression of *spo0A* could lead to an increase of iturin yield (Sun et al., 2021). This might be explained that overexpression of *spo0A* could cause interference to the expression of native *spo0A* in the wild-type strain. For this reason, overexpression of *spo0A* for increasing antifungal activity and lipopeptides production should be done in Δ*spo0A* rather than in WH1.

The aspartate phosphatase family RapA can indirectly dephosphorylate Spo0A∼P by dephosphorylating the phosphate group transporter Spo0F∼P (Reder et al., 2012), and the phosphatase Spo0E is able to directly dephosphorylate Spo0A∼P to Spo0A (Babel et al., 2020). Deletion of *spo0E* and *rapA* could both significantly increase the antifungal activity in WH1. Thereby, Spo0A is essential for biosynthesis of antifungal lipopeptides, and maintaining a certain level of Spo0A∼P is favorable for strengthening the antifungal activity in *B. amyloliquefaciens*, consistent with the previous reports that biosynthesis of fengycin and iturin is regulated by Spo0A in *B. subtilis* (Rahman et al., 2006; Zhang et al., 2016; Zhao et al., 2018). On this basis, we deleted *spo0E* and *rapA* in Δ*kinA*Δ*bdh*Δ*dhbF*, respectively, but only deletion of *rapA* could further increase the antifungal activity in Δ*kinA*Δ*bdh*Δ*dhbF*. This might be due to the reason that RapA is able to dephosphorylate several phosphorylated regulators, while Spo0E can only dephosphorylate Spo0A∼P (Rahman et al., 2006; Sun et al., 2021).

4-Phosphopantetheinyl transferase (Sfp) is essential for biosynthesis of lipopeptides in *B. subtilis* (Reuter et al., 1999; Wu et al., 2019; Yang et al., 2020). After deletion of *sfp*, the antifungal activity was significantly decreased in WH1. Thus, Sfp is also essential for biosynthesis of antifungal lipopeptides and showing antifungal activity in *B. amyloliquefaciens*. This result is consistent with the previous report in *B. subtilis* (Tan et al., 2022). Consistently, overexpression of *sfp* could further increase the antifungal activity in Δ*kinA*Δ*bdh*Δ*dhbF*Δ*rapA*.

The antifungal activity was increased step by step in the engineered strains, including Δ*kinA*, Δ*kinA*Δ*bdh*, Δ*kinA*Δ*bdh*Δ*dhbF*, Δ*kinA*Δ*bdh*Δ*dhbF*Δ*rapA* and Δ*kinA*Δ*bdh*Δ*dhbF*Δ*rapA*/T2-*sfp*. Finally, the titer of iturin achieved at 17.0 mg/L in Δ*kinA*Δ*bdh*Δ*dhbF*Δ*rapA*/T2-*sfp* with an increase of 3.2-fold compared to that of WH1. After fermentation optimization, the titer of iturin and fengycin achieved at 123.5 mg/L and 1200.8 mg/L in 50 L bioreactor, respectively. Compared to that of WH1, the final iturin and fengycin titer of Δ*kinA*Δ*bdh*Δ*dhbF*Δ*rapA*/T2-*sfp* increased by 22.8-fold and 15.9-fold, respectively. Dang et al. (2019) inserted a strong promoter at the upstream of *itu* operon to increase the iturin titer to 37.35 mg/L, but it was lower than our titer (123.5 mg/L) in this study. He et al. (2021) used *B. subtilis* 168 as a surrogate for improving fengycin production by overexpression of *accA* (encoding acetyl-CoA carboxylase), *cypC* (encoding fatty acid beta-hydroxylating cytochrome P450) and *gapA* (encoding glyceraldehyde-3-phosphate dehydrogenase). As a result, the final fengycin production reached 59.87 mg/L in the engineered *B. subtilis* 168, but it was also much lower than our fengycin production (1200.8 mg/L) in this study.

In conclusion, we have systematically developed a metabolically engineered cell factory to improve the antifungal activity and increase the antifungal lipopeptides production in *B. amyloliquefaciens*, such as blocking the carbon overflow metabolism to increase the supply of precursor branched-chain amino acids, deletion of *kinA* to disrupt sporulation for extending the stage to produce secondary metabolites, knock out of *dhbF* to hinder biosynthesis of siderophore for abolishing the competence of substrates, deletion of *rapA* to maintain an appropriate Spo0A∼P level, and overexpression of *sfp* to enhance the activation of substrates. In addition, we have also systematically revealed several metabolic pathways and regulators to directly or indirectly influence the antifungal activity and biosynthesis of iturin and fengycin in *B. amyloliquefaciens*. This work may open up a new avenue for the commercial production of iturin and fengycin in *B. amyloliquefaciens*.

## Data Availability

The original contributions presented in the study are included in the article/Supplementary Material, further inquiries can be directed to the corresponding author.
