# Supplementary material for: Systemically engineering Bacillus amyloliquefaciens for increasing its antifungal activity and green antifungal lipopeptides production
Source: Front Bioeng Biotechnol. 2022 Sep 7;10:961535. doi: 10.3389/fbioe.2022.961535 (PMC9490133; doi:10.3389/fbioe.2022.961535)
Supplement: Supplementary file 1 [file DataSheet1.docx]

**Supplementary materials**

1. ***Isolation and characterization of WH1***

WH1 was isolated from the rice (*Oryza sativa*) root located in Huazhong Agricultural University, Wuhan, China. Briefly, the rice roots were washed with water extensively, dipped in 70% (v/v) ethanol for 30 s, then treated with 0.1% (w/v) HgCl_2_ for 1 min. After that, the roots were washed with sterilized water, cut to fragments (~ 1 mm), then used for isolating endophytes on the PDA (potato dextrose agar) plates. One strain was isolated with excellent antifungal activity and named as WH1. It was characterized as *Bacillus amyloliquefaciens* by crystal violet staining, observation with transmission electronic microscope (TEM) and analysis of 16S rDNA sequence, and stored in China Center for Type Culture Collection (CCTCC NO: M208127).


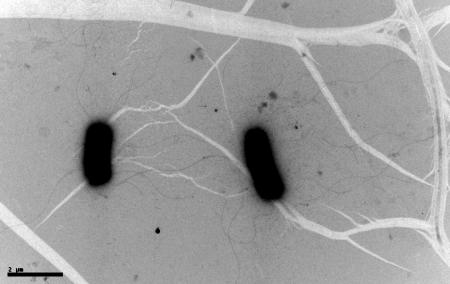

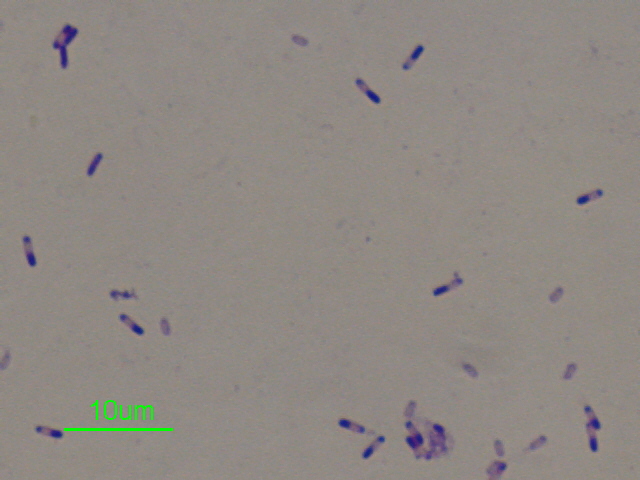


B

A


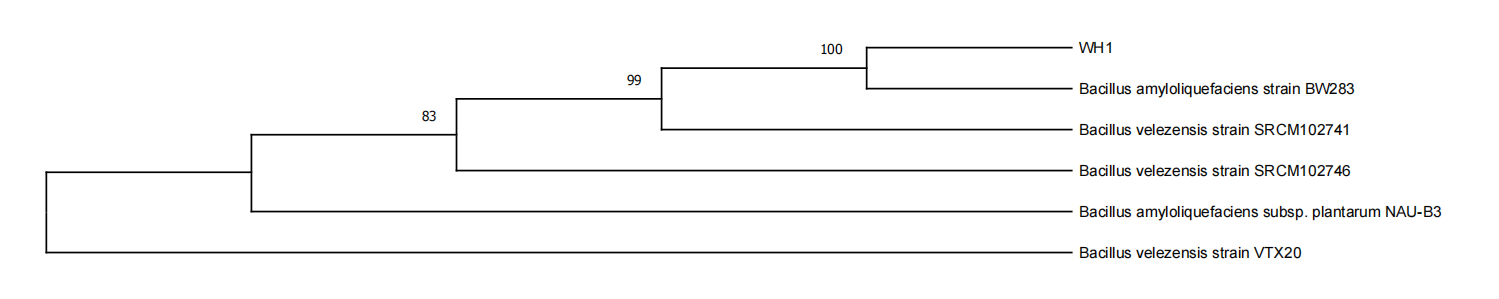


C

**Fig. S1 Characterization of WH1. A:** Observation by TEM. The size range of cells were 1 - 1.4 μm × 2.3 - 3.5 μm with flagellums. **B:** Spores. Crystal violet staining showed the spores of WH1. **C:** Phylogenetic tree of WH1 based on the 16S rDNA sequence.

1. ***Construction of knockout strains***

Two arms (~ 500 bp) homologous to the 5’ and 3’ coding regions of the targeted gene were amplified from *B. amyloliquefaciens* WH1 by PCR with the primers listed in Table S1, ligated by splicing with Overlapping Extension PCR (SOE-PCR), then subcloned into the vector T2(2)-ori with a temperature-sensitive replicon to promote single crossover. The constructed plasmids were used for transformation of WH1. Briefly, WH1 or other strains were incubated in LB medium at 28 ^o^C and 180 rpm overnight, then 50 μL culture was transferred into 5 mL fresh LB medium for further incubation. After 3 hours, 100 μL of culture (OD_600_ ≈ 0.3) was collected and mixed with 10 μL plasmid for incubation without shaking. After 1 h, the broth was further incubated with shaking at 180 rpm for 24 h, then 100 μL of culture was spread on LB agar plates with Kanamycin (20 µg/mL). After incubation for 2 days, the transformants were selected by Kanamycin resistance, then verified by PCR using LF and RR primers. PCR-selected transformants were cultured in LB medium with Kanamycin (20 μg/ml) at 45 ^o^C for 8 hours to promote the first crossover, then the mutants with single crossover were selected by PCR with the primers listed in Table S1. Selected colonies were picked up and cultured in LB medium at 28 ^o^C for 8 h, then the cells were spread on LB agar plates and replicated on Kanamycin plates for selecting sensitive colonies. Finally, the knockout strains were screened out that had looped out Kanamycin-resistant gene by the second crossover, and confirmed by PCR with the related primers LF and RR (Table S1) following with nucleotide sequencing of the PCR products (Qi et al., 2014).

**Table S1 Primers used in this study**

| Name | Sequence 5′→3′ | Purpose |
| --- | --- | --- |
| *bdh*-LF | CG**GGATCC**AATTATACACCAAGAAG | Amplifying L arm of *bdh* and verification of double-crossover |
| *bdh*-LR | TGATATTCACCATTTGTTCATCAAG | Amplifying L arm of *bdh* |
| *bdh*-RF | AACAAATGGTGAATATCAGACACAT | Amplifying R arm of *bdh* |
| *bdh*-RR | GC**TCTAGA**TTGACGGTGTTGATGTT | Amplifying R arm of *bdh* and verification of double-crossover |
| *bdh*-dan-LF | AGCACTTGCATCAGAAGAGT | Verification of single-crossover in *bdh* |
| *bdh*-dan-RR | AATGCTGTCCAGTTCCTTAG |  |
| *tnrA*-LF | CG**GGATCC**ATTGATTTCCGGAAGC | Amplifying L arm of *tnrA* and verification of double-crossover |
| *tnrA*-LR | AAGACATCCATTCAGCGTCGTAATCA | Amplifying L arm of *tnrA* |
| *tnrA*-RF | GACGCTGAATGGATGTCTTTTACT | Amplifying R arm of *tnrA* |
| *tnrA*-RR | GC**TCTAGA**TATAGGGAATGTTCCG | Amplifying R arm of *tnrA* and verification of double-crossover |
| *tnrA*-dan-LF | TGCTCCAAGACCAGGA | Verification of single-crossover in *tnrA* |
| *tnrA*-dan-RR | CCCATAAACAAAGACAGCAAGCGA |  |
| *codY*-LF | CG**GCATCC**ATATGGATGAAGTCG | Amplifying L arm of *codY* and verification of double-crossover |
| *codY*-LR | TTGTCCATCCTCCTAGAATTCCTC | Amplifying L arm of *codY* |
| *codY*-RF | TTCTAGGAGGATGGACAATAGGTGA | Amplifying R arm of *codY* |
| *codY*-RR | GC**TCTAGA**TTGAAAGCCACTCA | Amplifying R arm of *codY* and verification of double-crossover |
| *codY*-dan-LF | CG**GGATCC**GGAGGTATAAGA | Verification of single-crossover in *codY* |
| cod*Y*-dan-RR | TGGAAGTCTACGGTCATGCACCTC |  |
| *spo0E*-LF | CG**GGATCC**AAGATGAAAAAGAACG | Amplifying L arm of *spo0E* and verification of double-crossover |
| *spo0E*-LR | CTGTACGCGGTCGGGTTATTGAGC | Amplifying L arm of *spo0E* |
| *spo0E*-RF | GCTCAATAACCCGACCGCGTACAG | Amplifying R arm of *spo0E* |
| *spo0E*-RR | GC**TCTAGA**CGCTCAAGGTCAACTG | Amplifying R arm of *spo0E* and verification of double-crossover |
| *spo0E*-dan-LF | CGAAAAGAATACGCTCATTGAAAA | Verification of single crossover in *spo0E* |
| *spo0E*-dan-RR | TGAACGGTTGGAAGTGAACTCGCT |  |
| *rapA*-LF | CG**GGATCC**AGTCAGGTCAATTTCG | Amplifying L arm of *rapA* and verification of double-crossover |
| *rapA*-LR | CGCTTGGTTTTCTCCCCCCTTTTG | Amplifying L arm of *rapA* |
| *rapA*-RF | CAAAAGGGGGGAGAAAACCAAGCG | Amplifying R arm of *rapA* |
| *rapA*-RR | GC**TCTAGA**AATTCCCTGGATCACT | Amplifying R arm of *rapA* and verification of double-crossover |
| *rapA*-dan-LF | AGAACAAAAAAATCCGCCTGCCTG | Verification of single-crossover in *rapA* |
| *rapA*-dan-RR | GCAAACGCAATCGCCAGTTCTTCT |  |
| *dhbF*-LF | CG**GGATCC**GGCTTTCGCCCATATA | Amplifying L arm of *dhbF* and verification of double-crossover |
| *dhbF*-LR | GATACACAACAGCTCTTCCTTCCG | Amplifying L arm of *dhbF* |
| *dhbF*-RF | CGGAAGGAAGAGCTGTTGTGTATC | Amplifying R arm of *dhbF* |
| *dhbF*-RR | GC**TCTAGA**GCCATCATGCGTGAAAGC | Amplifying R arm of *dhbF* and verification of double-crossover |
| *dhbF*-dan-LF | GTGAATGCTTTGCTAAGGAGTGCA | Verification of single-crossover in *dhb*F |
| *dhbF*-dan-RR | GC**TCTAGA**CCGCCGACAACAAG |  |
| *sfp*-LF | CG**GGATCC**TATCTTAAACATGGTG | Amplifying L arm of *sfp* and verification of double-crossover |
| *sfp*-LR | CTTTTTCTATGCGTCCAGATCCTC | Amplifying L arm of *sfp* |
| *sfp*-RF | GAGGATCTGGACGCATAGAAAAAG | Amplifying R arm of *sfp* |
| *sfp*-RR | GC**TCTAGA**CATTCAGGAATCCATC | Amplifying R arm of *sfp* and verification of double-crossover |
| *sfp*-dan-LF | GCATTTCGGGCTTTCTGTCG | Verification of single-crossover in *sfp* |
| *sfp*-dan-RR | GCCGGCAAAGAGATTGTCGT |  |
| *comK-*LF | CG**GGATCC**TCAAGCGCCTTATTAA | Amplifying L arm of *comK* and verification of double-crossover |
| *comK*-LR | CATCACAGCTTTGCCTCCAATCCT | Amplifying L arm of *comK* |
| *comK*-RF | AGGATTGGAGGCAAAGCTGTGATG | Amplifying R arm of *comK* |
| *comK*-RR | GC**TCTAGA**ACGTCCGCTAACGCCAAAA | Amplifying R arm of *comK* and verification of double-crossover |
| *comK*-dan-LF | AGACGATTCA TGTGAACGGC | Verification of single-crossover in *comK* |
| *comK*-dan-RR | GTCAGGGCTGTCGATACTTG |  |
| *sigD*-LF | CG**GGATCC**ACTTCTATTTTTGACC | Amplifying L arm of *sigD* and verification of double-crossover |
| *sigD*-LR | TCACTCGCTAACTGTTATCCCCCT | Amplifying L arm of *sigD* |
| *sigD*-RF | AGGGGGATAACAGTTAGCGAGTGA | Amplifying R arm of *sigD* |
| *sigD*-RR | GC**TCTAGA**CACAGCTTTATGCGAC | Amplifying R arm of *sigD* and verification of double-crossover |
| *sigD*-dan-LF | AGGCTCCTATTTAACGGCATTGG | Verification of single-crossover in *sigD* |
| *sigD*-dan-RR | CTCCGCTTAGGTCATTTTTGTGC |  |
| *spo0A*-hF | CG**GGATCC**GGTTTCCATTGTTTTC | Amplifying *spo0A* gene for expression |
| *spo0A*-hR | GC**TCTAGA**TTCTTCACTTCTTTCC |  |
| *sfp-F* | TCGATGTATACTCAGCTCCG | Amplifying *sfp* gene for expression |
| *sfp-R* | TCCATATCACAAAAGCCTCC |  |

Restriction sites of *BamH* I and *Xba* I are indicated in bold.

1. ***Primers used in qRT-PCR***

**Table S2 Primers used in qRT-PCR**

| Name | Sequence 5′→3′ |
| --- | --- |
| 16s-qF | CTGCCTGTAAGACTGGGATAAC |
| 16s-qR | CATCTGTAAGTGGTAGCCGAAG |
| *ywaA*-qF | TAGCGGCATCCCATACGTACAA |
| *ywaA*-qR | GCTTTCAGGCTTGATGCGTAGT |
| *leuA*-qF | ATGTGCGAAAGCGTTATCTCCGA |
| *leuA*-qR | GCATAGGAGAAAGAGCCGGGAAC |

1. ***Antifungal activity of lipopeptides gene cluster mutant strains***

For confirming the relationship between lipopeptides and antifungal activity, we constructed a double knockout strain ∆*ituB*∆*fenA* on the basis of ∆*ituB* and ∆*fenA* (Chen et al., 2020). The antifungal activity was detected for WH1, ∆*ituB*, ∆*fenA* and ∆*ituB*∆*fenA*. It was found that both ∆*ituB* and ∆*fenA* showed a slightly weaker antifungal activity than the wild-type strain WH1, while ∆*ituB*∆*fenA* almost lost the antifungal activity against *F. oxysporum* (Fig. S2). The results clearly showed that iturin and fengycin contribute most of the antifungal activity to *B.* *amyloliquefaciens* WH1. Thereby, we used the antifungal activity to asses the antifungal lipopeptides (iturin and fengycin) production in this study.

**
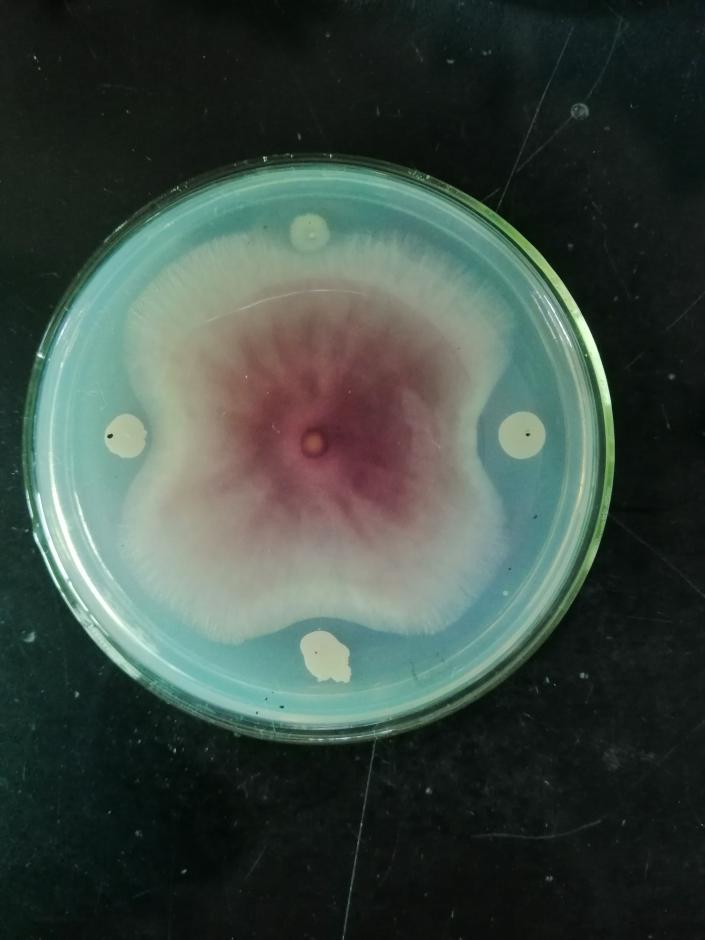
**

∆*ituB*

∆*fenA*∆*ituB*

∆*fenA*

WH1

**Fig. S2 Antifungal activity of lipopeptides gene cluster mutant strains.**

1. ***Optimization of fermentation medium***

*5.1 Methods*

The engineered strain Δ*kinA*Δ*bdh*Δ*dhbF*Δ*rapA*/T2-*sfp* was cultured in the mediums including LB (yeast extract 5.0 g, peptone 10.0 g, NaCl 10.0 g in 1.0 L distilled water, pH 7.0), NB (peptone 5.0 g, meat extracts 3.0 g, glucose 2.5 g in 1.0 L distilled water, pH7.0) (Horsburgh & Moir 1999), BPY (glucose 5.0 g, meat extracts 5.0 g, peptone 10.0 g, yeast extract 5.0 g, NaCl 5.0 g in 1.0 L distilled water, pH7.0) (Zhang et al., 2016), Landy (glucose 20.0 g, L-sodium glutamate 5.0 g, yeast extract 1.0 g, MgSO_4_ 0.5 g, KCl 0.5 g, KH_2_PO_4_ 1.0 g, FeSO_4_ 0.15 mg, MnSO_4_ 5.0 mg, CuSO_4_ 0.16 mg in 1.0 L distilled water, pH7.0) (Li et al., 2022) and modified Landy (glucose 15.0 g, L-sodium glutamate 6.0 g, MgSO_4_ 0.2g, MnSO_4_ 2.0 mg, KH_2_PO_4_ 0.5 g in 1.0 L distilled water, pH 7.0) (Fu et al., 2022) at 37 ^o^C for 48 h, then the antifungal activity of broth was determined. It was found that modified Landy medium was more favorable for producing antifungal lipopeptides, thereby it was selected for further optimization.

First, we optimized carbon sources for producing antifungal lipopeptides. Glucose, dextrin, glycerol, sucrose, corn flour and soluble starch were used as carbon source at a concentration of 5.0, 10.0, 15.0, 20.0 and 25.0 g/L respectively. After being cultured at 37 ^o^C for 48 h, the antifungal activity of broth supernatant was determined respectively.

Second, we optimized nitrogen sources for producing antifungal lipopeptides. The inorganic nitrogen sources including NaNO_3_ and (NH_4_)_2_SO_4_, and organic nitrogen sources including peptone, yeast powder, soybean flour, soybean meal powder and fish meal power were used for producing antifungal lipopeptides at a concentration of 5.0, 10.0, 15.0, 20.0 and 25.0 g/L respectively. After being cultured at 37 ^o^C for 48 h, the antifungal activity of broth supernatant was determined respectively.

Third, we optimized inorganic salts, including MgSO_4_•7H_2_O (0, 0.25, 0.50, 0.75, 1.00 and 1.25 g/L respectively), CaCl_2_ (0, 0.2, 0.4, 0.6, 0.8 and 1.0 g/L respectively), ZnSO_4_•7H_2_O (0, 0.2, 0.4, 0.6, 0.8 and 1.0 g/L respectively), KH_2_PO_4_ (0, 0.25, 0.50, 0.75, 1.00 and 1.25 g/L respectively), MnSO_4_•H_2_O (0, 1.0, 3.0, 5.0, 7.0 and 10.0 mg/L respectively), FeSO_4_•7H_2_O (0, 0.5, 1.0, 1.5, 2.0, 2.5 mg/L respectively) for producing antifungal lipopeptides. After being cultured at 37 ^o^C for 48 h, the antifungal activity of broth supernatant was determined respectively.

Finally, we optimized amino acids for producing antifungal lipopeptides. L-Ile, L-Ser, L-Pro and L-Glu were used at a concentration of 0, 0.2, 0.5, 0.8, 1.0 g/L respectively. After being cultured at 37 ^o^C for 48 h, the antifungal activity of broth supernatant was determined respectively.

On the basis of above experiments, the orthogonal test was set up for further optimizing the fermentation medium. Glucose, soybean meal power, MgSO_4_•7H_2_O, ZnSO_4_•7H_2_O and KH_2_PO_4_ were selected for further optimization according to the orthogonal experiment L_16_(4^5^) (Table S3).

**Table S3 Factorial Level**

| Factorial level | Glucose (g/L) | Soybean meal power (g/L) | MgSO_4_•7H_2_O  (g/L) | ZnSO_4_•7H_2_O  (g/L) | KH_2_PO_4_  (g/L) |
| --- | --- | --- | --- | --- | --- |
| 1 | 15 | 15 | 0.5 | 0.2 | 0.5 |
| 2 | 15 | 20 | 0.75 | 0.4 | 0.75 |
| 3 | 15 | 25 | 1.0 | 0.6 | 1.0 |
| 4 | 15 | 30 | 1.25 | 0.8 | 1.25 |

*5.2 Results*

We used five medium including LB, NB, BPY, Landy and modified Landy, for culturing Δ*kinA*Δ*bdh*Δ*dhbF*Δ*rapA*/T2-*sfp* to produce antifungal lipopeptides. It was found the antifungal activity was higher in the broth cultured with modified Landy medium, and the OD_600_ was higher in the broth cultured with LB medium (Fig. S3).

**
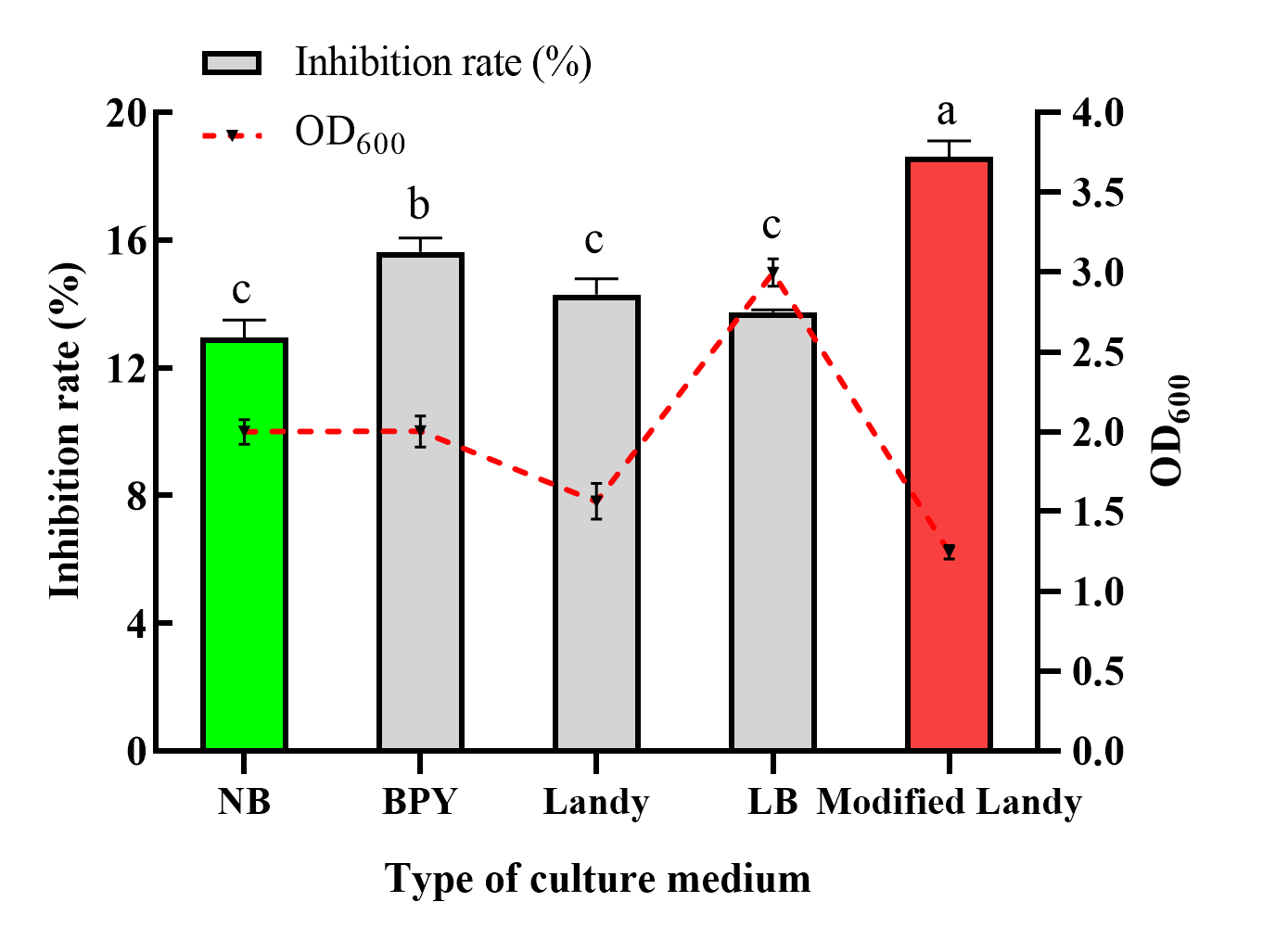
**

**Fig. S3 Influence of media on inhibition rate and OD_600_ of ΔkinAΔbdhΔdhbFΔrapA/T2-sfp.**

On the basis of modified Landy medium, we further optimized the medium including carbon sources, nitrogen sources, etc. Glucose, dextrin, glycerol, sucrose, corn flour and soluble starch were used as carbon source for culturing Δ*kinA*Δ*bdh*Δ*dhbF*Δ*rapA*/T2-*sfp*, then the antifungal activity of broth was determined respectively. The results showed that glucose was favorable for increasing both of the biomass and antifungal activity (Table S4). When the concentration of glucose was 20 g/L, the inhibition rate of broth achieved at the maximum value of 27% (Fig. S4).

**Table S4 Effects of carbon source and concentration on the inhibition rate (%) of Δ*kinA*Δ*bdh*Δ*dhbF*Δ*rapA*/T2-*sfp***

| Concentration(g/L) | Glucose | Dextrin | Sucrose | Glycerol | Corn flour | Soluble starch |
| --- | --- | --- | --- | --- | --- | --- |
| 5 | 9.78±0.99 | 18.75±0.09 | 9.94±0.47 | 14.25±0.90 | 15.92±0.90 | 19.55±0.54 |
| 10 | 19.83±1.02 | 22.16±0.11 | 20.74±0.55 | 7.54±0.87 | 18.99±0.45 | 19.83±0.44 |
| 15 | 25.14±0.12 | 24.43±0.45 | 23.86±0.86 | 4.47±0.50 | 20.11±0.10 | 21.78±0.11 |
| 20 | **27.37±0.43** | 26.13±0.44 | 26.42±0.13 | 2.51±0.01 | 22.35±0.59 | 23.47±0.94 |
| 25 | 25.70±0.44 | 25.00±1.39 | 25.85±0.57 | 1.96±0.49 | 17.04±0.53 | 21.79±0.11 |


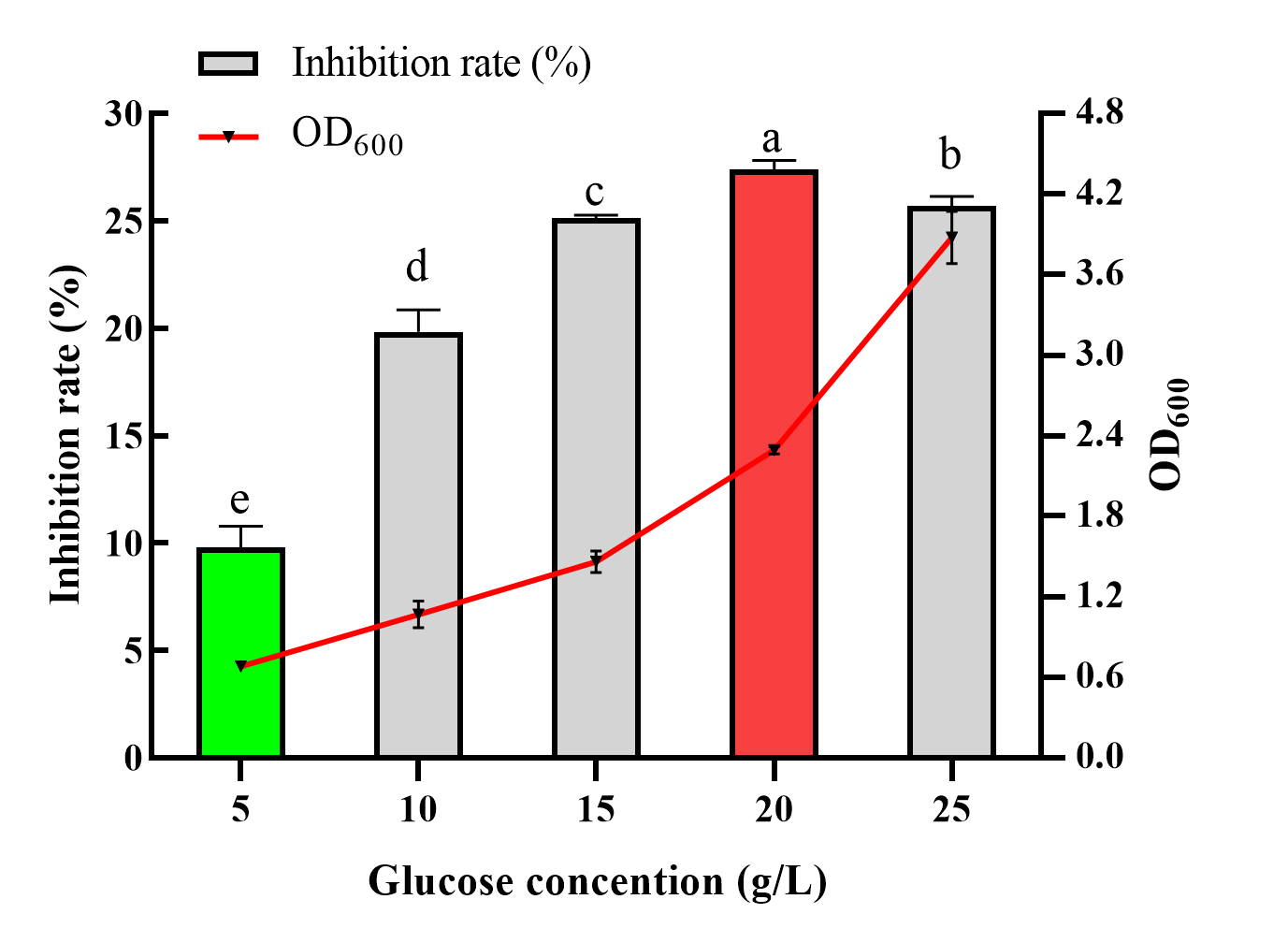


**Fig. S4 Effects of glucose concentration on the inhibition rate and biomass (OD_600_) of Δ*kin*AΔ*bdh*Δ*dhbF*Δ*rapA*/T2-*sfp*.**

The inorganic nitrogen sources including NaNO_3_ and (NH_4_)_2_SO_4_, and organic nitrogen sources including peptone, yeast powder, soybean flour, soybean meal powder and fish meal power were determined for producing antifungal lipopeptides. The results showed that soybean meal powder was more efficient for producing antifungal lipopeptides than other nitrogen sources (Table S5). When the concentration of soybean meal power was 20 g/L, the inhibition rate of broth achieved at the maximum value of 34% (Fig. S5). It has been reported that nitrogen sources have significant influences on the synthesis of iturin (Yang et al., 2020), consistent with our results in this study. However, excessive nitrogen source tends to terminate the synthesis of iturin A (Yang et al., 2020), also consistent with our results in this study.

**Table S5 Effects of nitrogen source and concentration on the inhibition rate (%) of Δ*kinA*Δ*bdh*Δ*dhbF*Δ*rapA*/T2-*sfp***

| Concentration  (g/L) | Sodium nitrate | Ammonium sulfate | Peptone | Yeast powder | Soybean cake powder | Soybean meal powder | Fish meal |
| --- | --- | --- | --- | --- | --- | --- | --- |
| 5 | 16.05±0.37 | 23.78±0.54 | 29.19±0.33 | 25.14±0.11 | 26.01±0.11 | 30.64±0.23 | 1.44±0.63 |
| 10 | 10.89±0.39 | 22.06±0.35 | 27.17±0.22 | 26.30±0.22 | 30.06±0.32 | 31.5±0.24 | 3.18±0.43 |
| 15 | 2.58±0.01 | 22.07±0.53 | 27.46±0.33 | 5.78±0.42 | 19.66±0.53 | 32.08±0.41 | 3.47±0.49 |
| 20 | 2.00±0.43 | 22.35±0.10 | 25.72±0.34 | 4.34±0.02 | 17.34±0.57 | **34.68±0.4** | 6.65±0.41 |
| 25 | 1.72±0.01 | 20.63±0.10 | 25.43±0.22 | 2.02±0.17 | 16.76±0.37 | 31.50±0.24 | 1.44±0.44 |


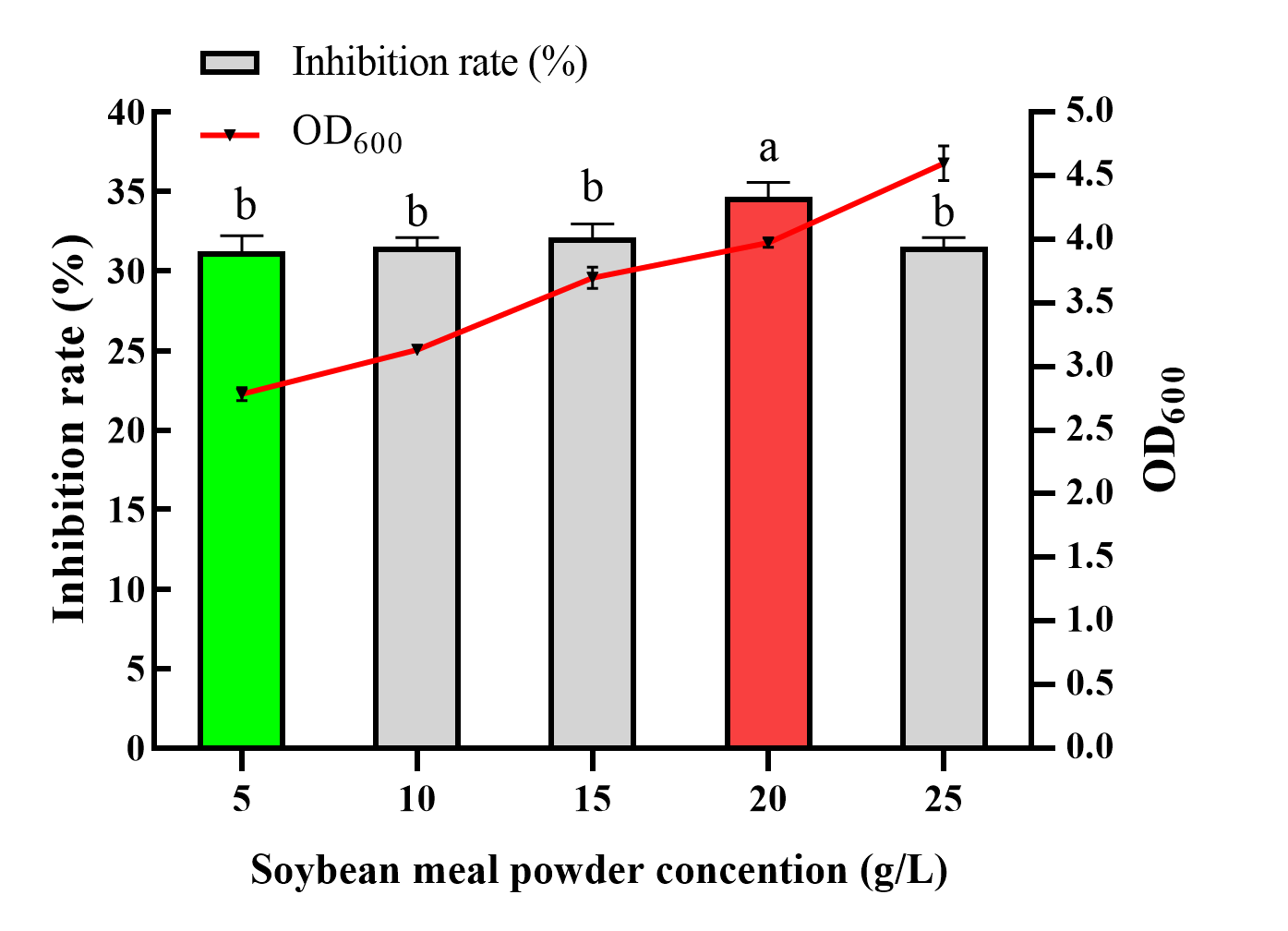


**Fig. S5 Effects of soybean meal powder concentration on the inhibition rate and biomass (OD_600_) of Δ*kin*AΔ*bdh*Δ*dhbF*Δ*rapA*/T2-*sfp*.**

We further optimized inorganic ions including Mg^2+^, K^+^, Zn^2+^, Mn^2+^, Fe^2+^ and Ca^2+^ for producing antifungal lipopeptides. It was found that MgSO_4_•7H2O at 0.75 g/L (Fig. S6A), KH_2_PO_4_ at 1.0 g/L (Fig. S6C), ZnSO_4_•7H_2_O at 0.6 g/L (Fig. S6D) and MnSO_4_•H_2_O at 7.0 mg/L (Fig. S6F) could all significantly increase the antifungal activity in broth. However, CaCl_2_ (Fig. S6B) and FeSO_4_ (Fig. S6E) had no significant influence on the antifungal activity of broth.

A B


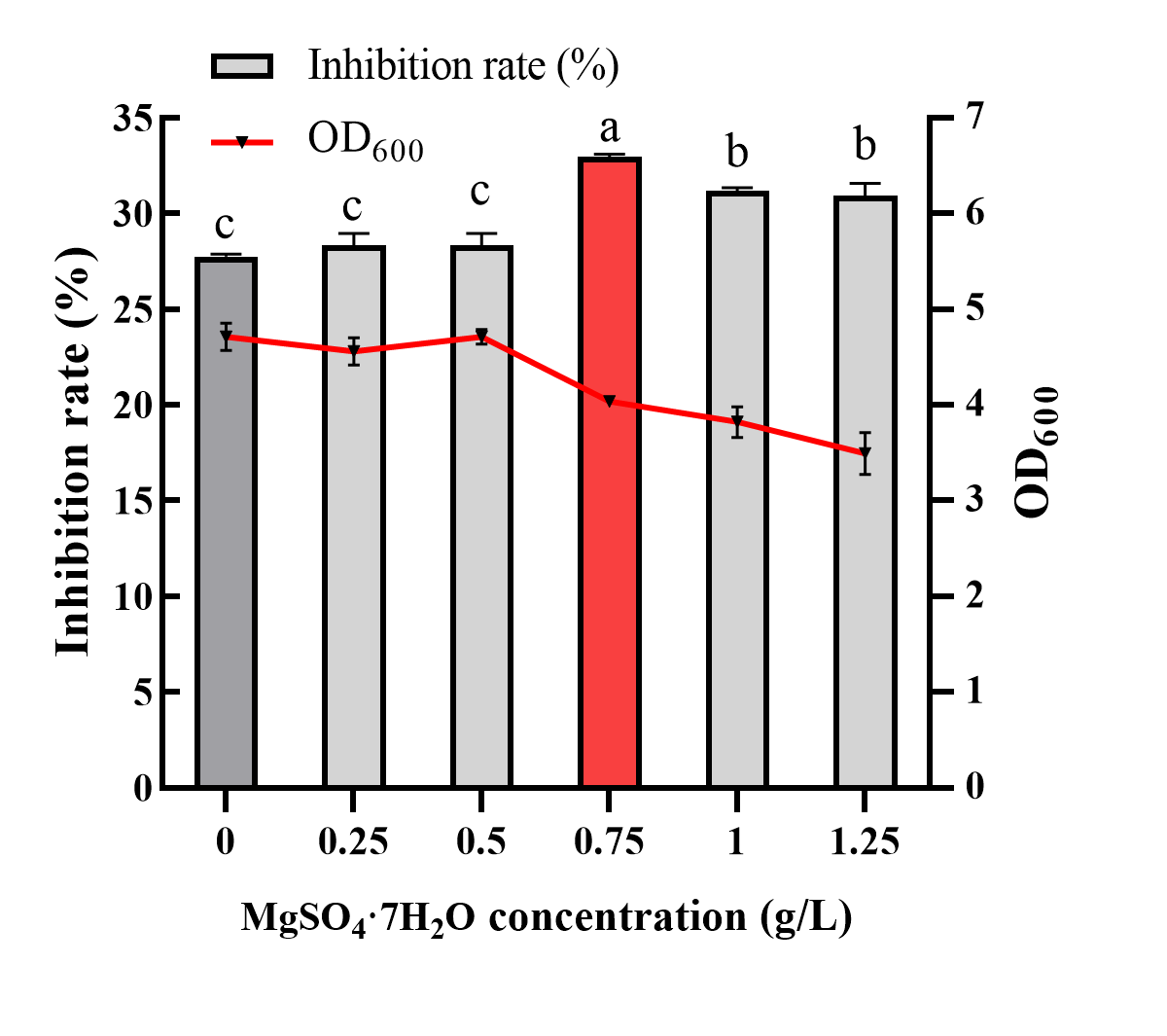

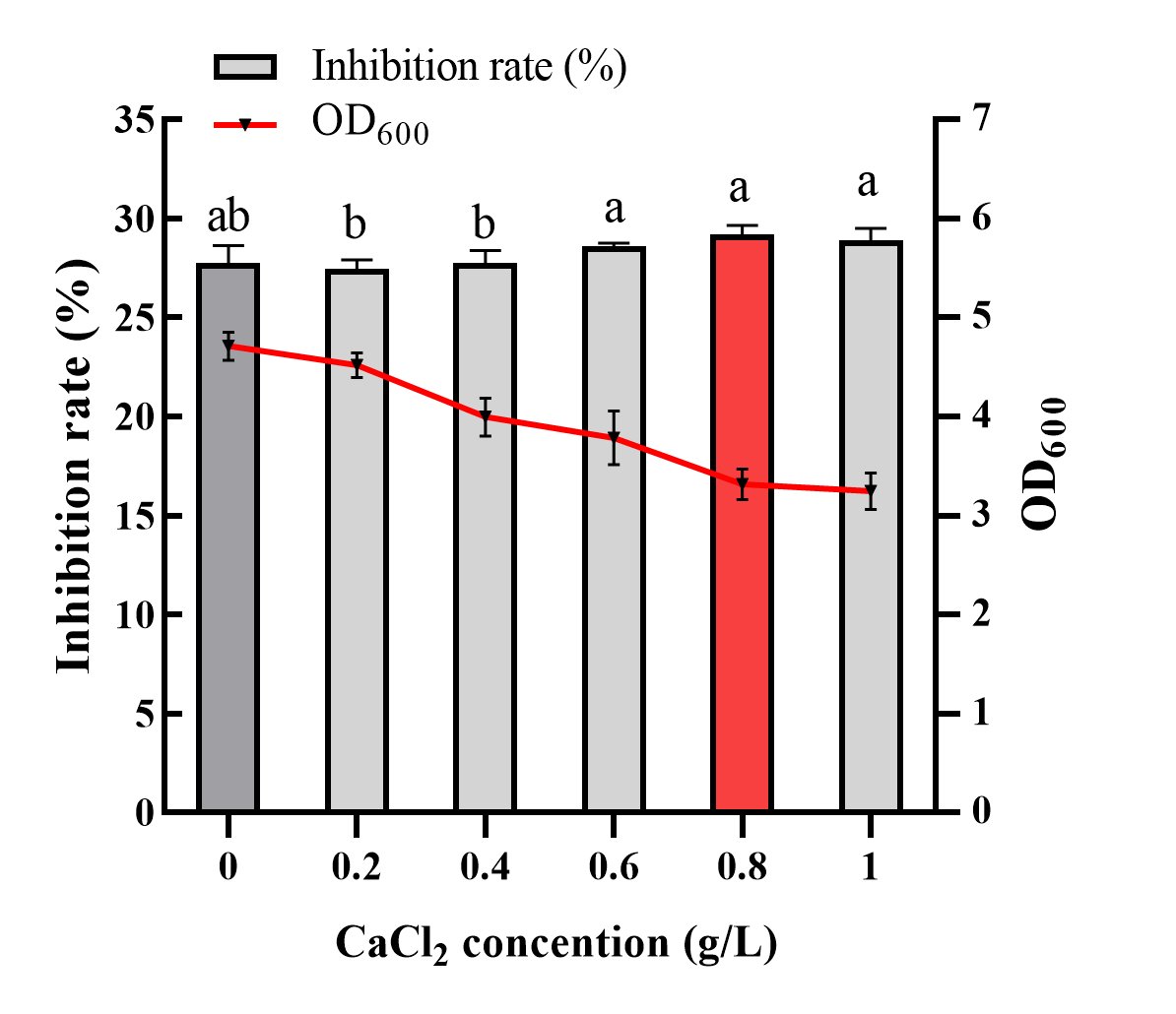


C D


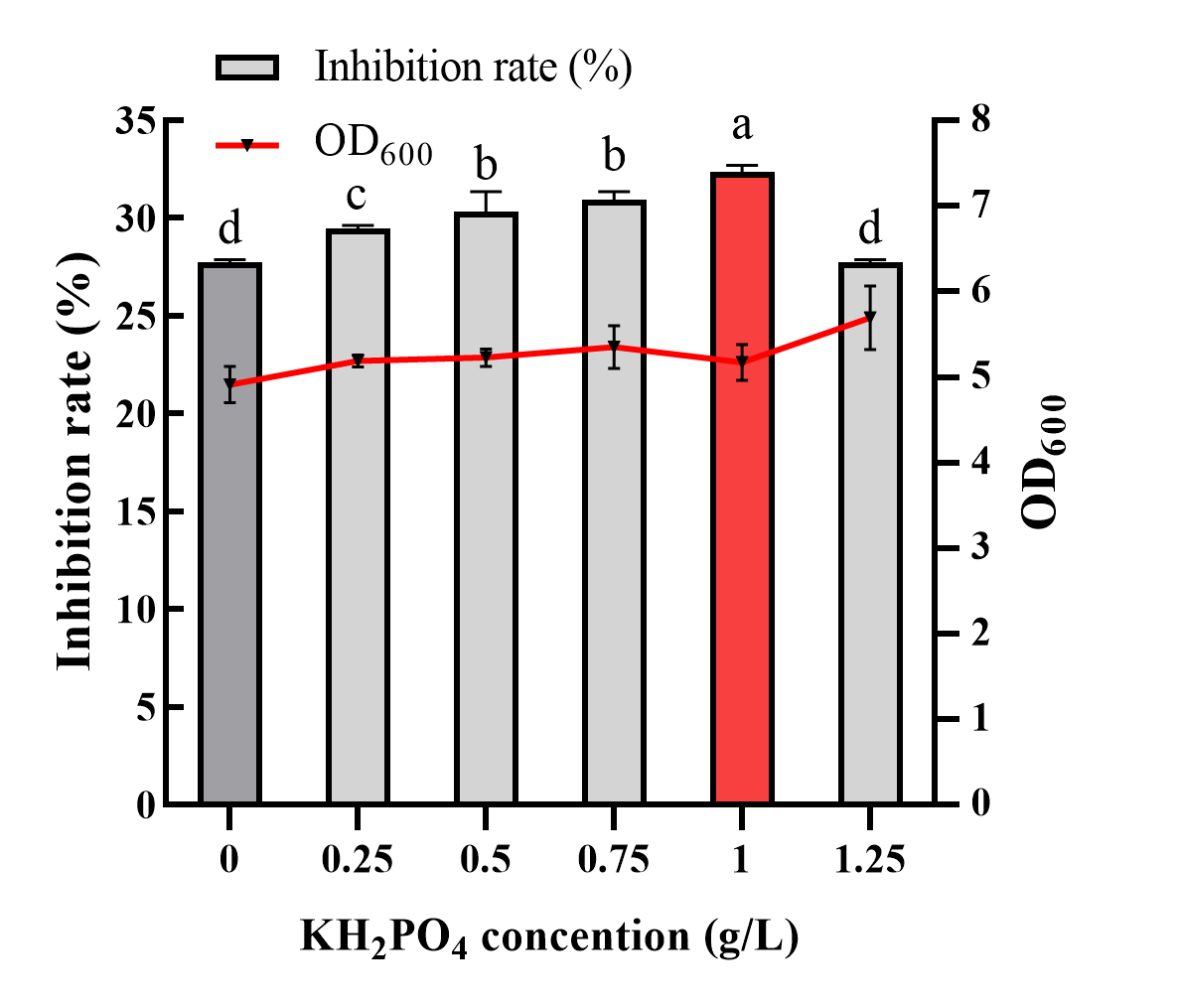

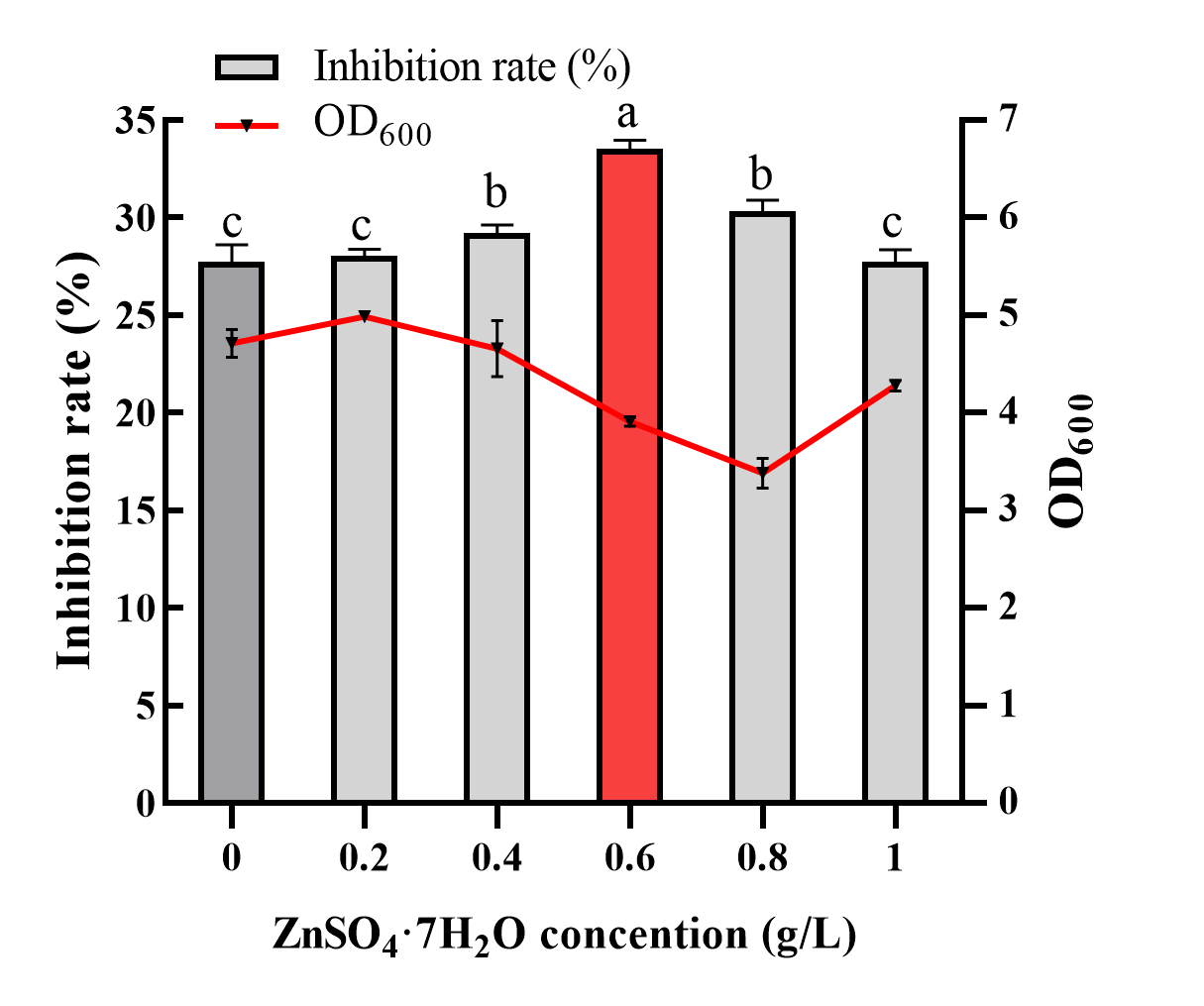


E F


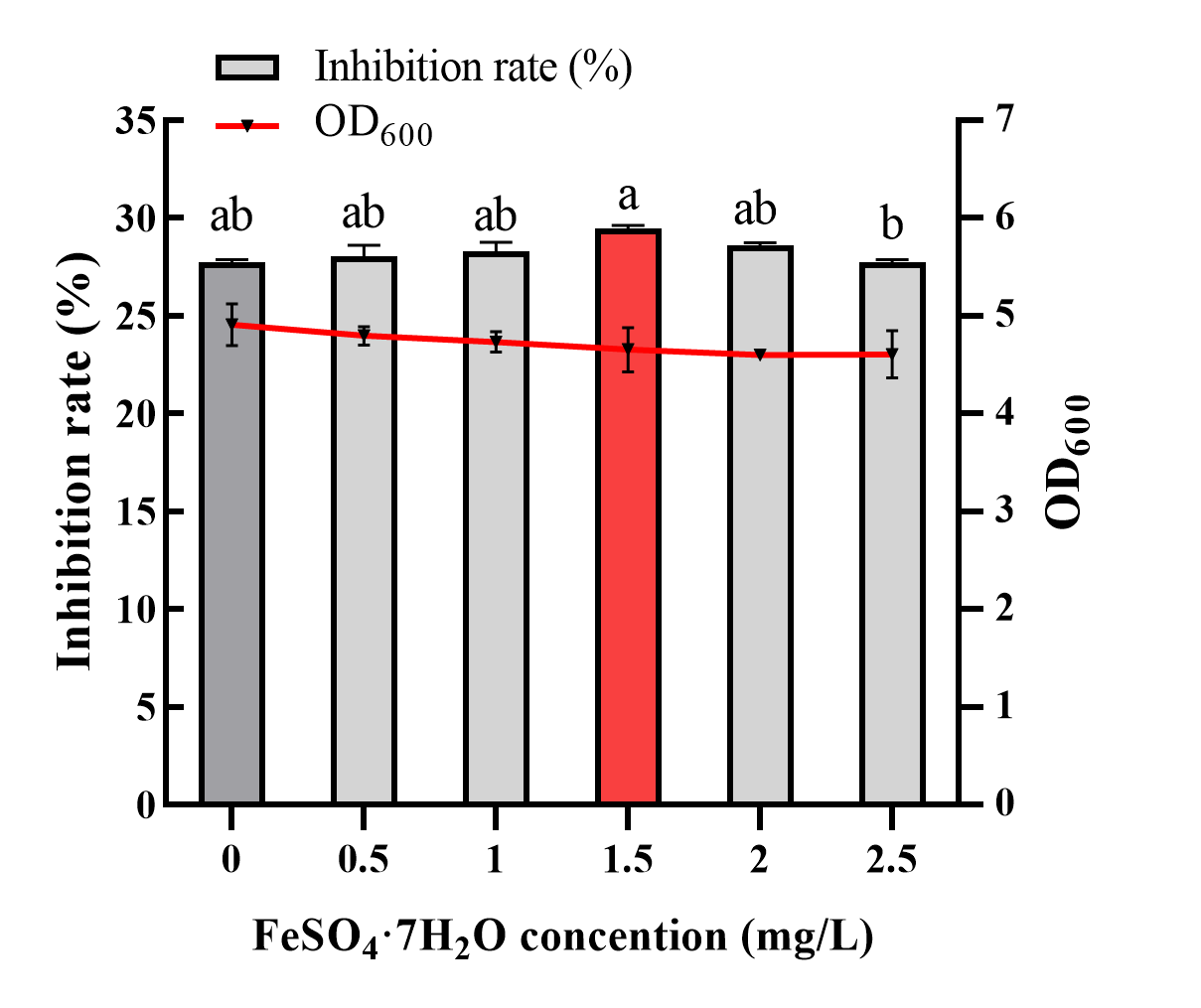

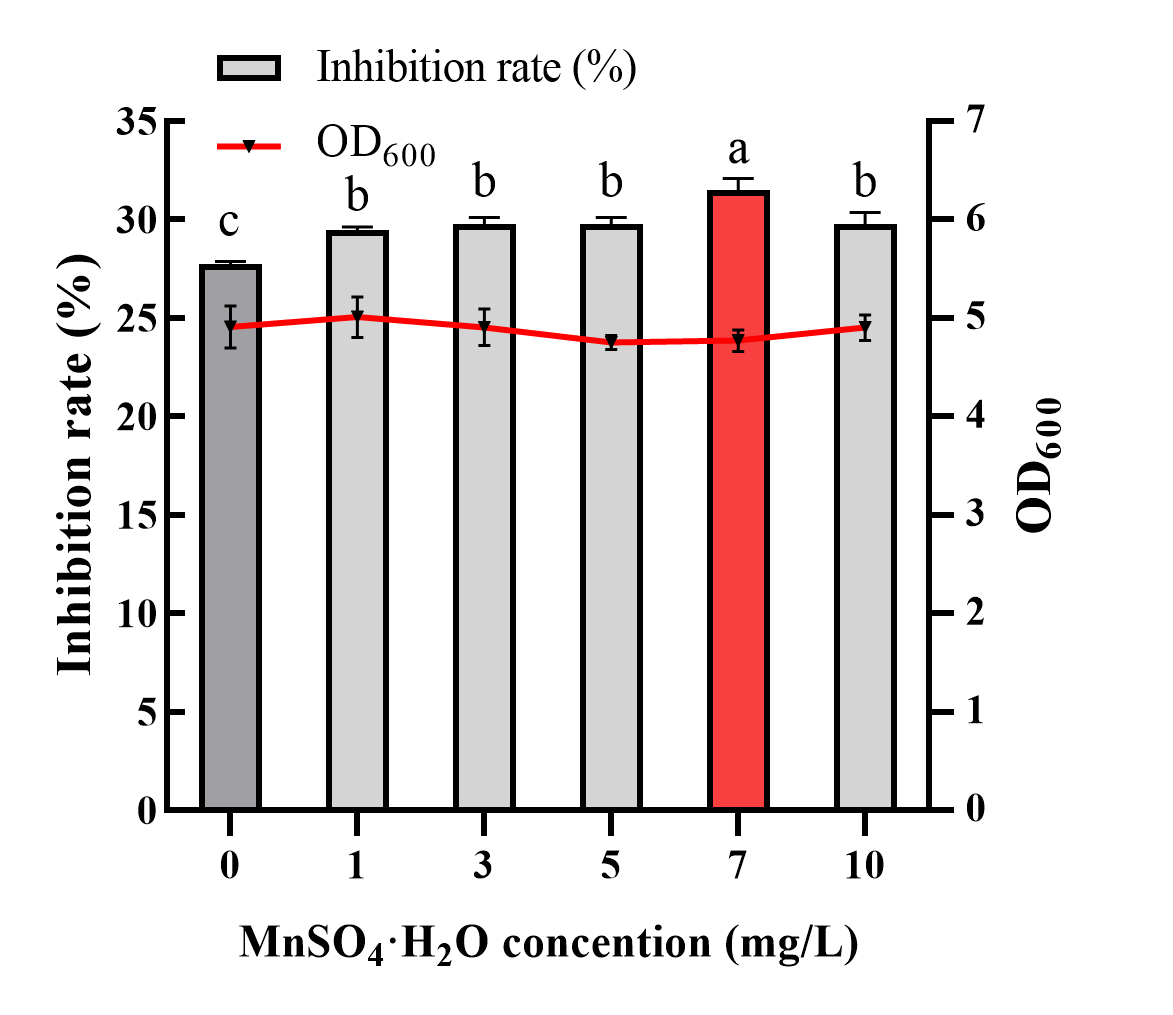


**Fig. S6 Effects of inorganic salts on the inhibition rate and biomass (OD_600_) of Δ*kin*AΔ*bdh*Δ*dhbF*Δ*rapA*/T2-*sfp*. A:** MgSO_4_·7H_2_O; **B:** CaCl_2_: **C:** K_2_HPO_4;_ **D:** ZnSO_4_·7H_2_O; **E:** FeSO_4_·7H_2_O; **F:** MnSO_4_·H_2_O.

We selected four amino acids including L-Ile, L-Ser, L-Pro and L-Glu, which are substrates for biosynthesis of antifungal lipopeptides, for culturing Δ*kinA*Δ*bdh*Δ*dhbF*Δ*rapA*/T2-*sfp* to produce iturin and fengycin. The results showed that addition with these amino acids could not further improve the antifungal activity. Conversely, these amino acids, in particular L-Ser, led to a decrease of antifungal activity (Fig. S7).


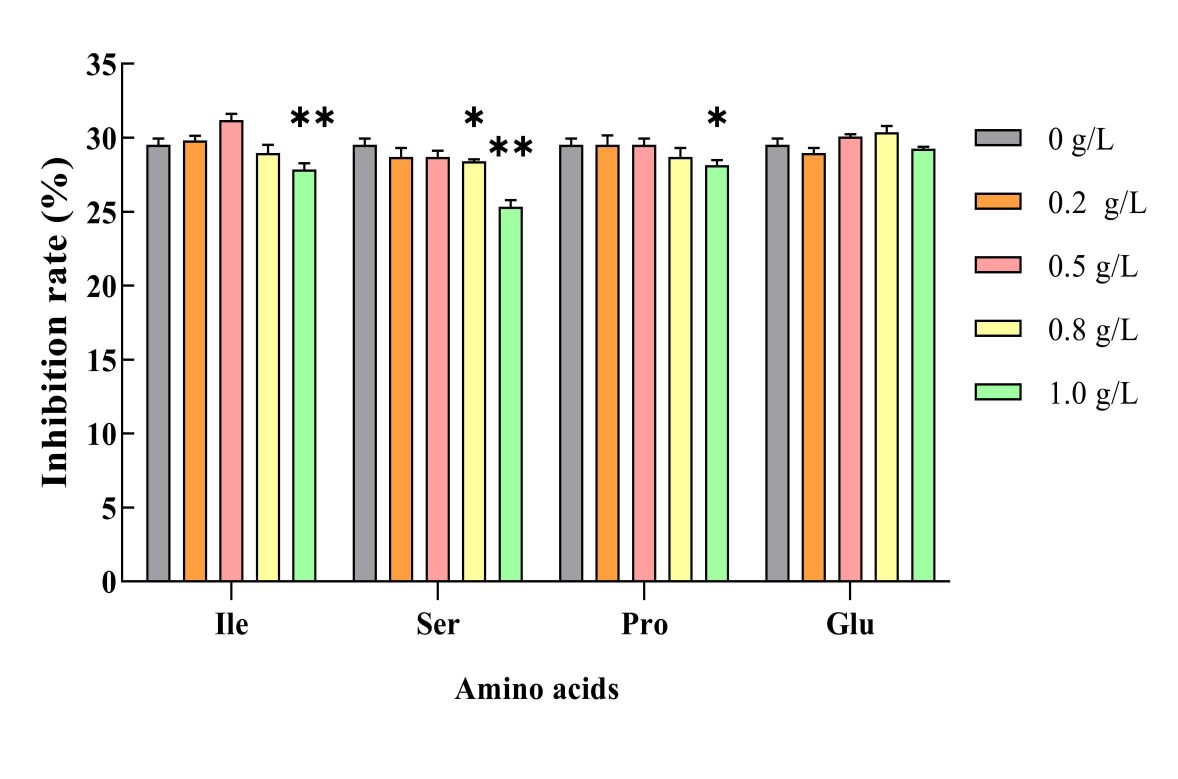


**Fig. S7 Effects of amino acids on the inhibition rate of Δ*kin*AΔ*bdh*Δ*dhbF*Δ*rapA*/T2-*sfp*.**

On the basis of above results, glucose, soybean meal power, MgSO_4_•7H_2_O, ZnSO_4_•7H_2_O and KH_2_PO_4_ were selected for further optimization according to the orthogonal experiment L_16_(4^5^). As shown in Table S6, according to R value obtained from the orthogonal tests, we found that the antifungal activity was successively affected by glucose, ZnSO_4_•7H_2_O, MgSO_4_•7H_2_O, soybean meal power and KH_2_PO_4_. The optimal combination of medium contains 20 g/L glucose, 20 g/L soybean meal power, 0.5 g/L MgSO4•7H2O, 1.0 g/L KH2PO4, 0.8 g/L ZnSO4•7H2O and 7.0 mg/L MnSO4•H2O. In this formula, the antifungal activity of broth achieved at 37.18%.

**Table S6 Results of Orthogonal experiment**

| Treatment | Glucose  (g/L) | Soybean  meal power (g/L) | MgSO_4_•H_2_O(g/L) | KH_2_PO_4_(g/L) | ZnSO_4_•7H_2_O  (g/L) | Antifungal  activity (%) |
| --- | --- | --- | --- | --- | --- | --- |
| 1 | 15 | 15 | 0.5 | 0.5 | 0.2 | 27.67 |
| 2 | 15 | 20 | 0.75 | 0.75 | 0.4 | 27.95 |
| 3 | 15 | 25 | 1.0 | 1.0 | 0.6 | 27.38 |
| 4 | 15 | 30 | 1.25 | 1.25 | 0.8 | 29.11 |
| 5 | 20 | 15 | 0.75 | 1.25 | 0.6 | 32.28 |
| **6** | **20** | **20** | **0.5** | **1.0** | **0.8** | **37.18** |
| 7 | 20 | 25 | 1.25 | 0.75 | 0.2 | 29.98 |
| 8 | 20 | 30 | 1.0 | 0.5 | 0.4 | 28.82 |
| 9 | 25 | 15 | 1.0 | 0.75 | 0.8 | 34.87 |
| 10 | 25 | 20 | 1.25 | 0.5 | 0.6 | 29.69 |
| 11 | 25 | 25 | 0.5 | 1.25 | 0.4 | 27.09 |
| 12 | 25 | 30 | 0.75 | 1.0 | 0.2 | 27.67 |
| 13 | 30 | 15 | 1.25 | 1.0 | 0.4 | 20.17 |
| 14 | 30 | 20 | 1.0 | 1.25 | 0.2 | 22.77 |
| 15 | 30 | 25 | 0.75 | 0.5 | 0.8 | 24.21 |
| 16 | 30 | 30 | 0.5 | 0.75 | 0.6 | 22.77 |
| k1 | 28.03 | 28.75 | 28.53 | 27.60 | 27.03 |  |
| k2 | 32.07 | 29.40 | 28.75 | 28.89 | 26.01 |  |
| k3 | 29.83 | 27.17 | 28.46 | 28.10 | 28.03 |  |
| k4 | 22.48 | 27.09 | 27.24 | 27.81 | 31.34 |  |
| R | 9.59 | 2.31 | 1.51 | 1.29 | 5.33 | Rang analysis |

*6. Optimization of fermentation conditions*

*6.1 Methods*

To study the impact of culture temperature on antifungal lipopeptides production, Δ*kinA*Δ*bdh*Δ*dhbF*Δ*rapA*/T2-*sfp* was cultured at 28, 32, 37 and 42 ^o^C respectively. To study the impact of pH on antifungal lipopeptides production, the initial pH value of medium was set at 6.0, 7.0, 7.3, 7.5, 8.0 and 9.0, respectively. The amounts of inoculation were set at 1%, 2%, 3%, 4%, and 5% (v/v) for culturing Δ*kinA*Δ*bdh*Δ*dhbF*Δ*rapA*/T2-*sfp* to produce antifungal lipopeptides respectively. To study the impact of ventilation on antifungal lipopeptides production, 250 mL flasks containing 50, 75, 100 and 125 mL medium, were prepared for culturing Δ*kinA*Δ*bdh*Δ*dhbF*Δ*rapA*/T2-*sfp*, respectively. All other factors were held constantly.

*6.2 Results*

With the optimized fermentation medium formula, we detected the effect of fermentation conditions on the antifungal lipopeptides production in Δ*kinA*Δ*bdh*Δ*dhbF*Δ*rapA*/T2-*sfp*. As shown in Fig. S8A, the culture temperature was selected as 37 ^o^C. At this temperature, the antifungal activity was higher than others. The effect of pH on antifungal activity was determined. It was found that pH 8.0 was more favorable for production of antifungal lipopeptides (Fig. S8B). The inoculation rate was detected for the antifungal lipopeptides production. At an inoculation rate of 2%, the antifungal activity of broth reached the maximum value (Fig. S8C). We also investigated the effect of liquid volume on antifungal lipopeptides production, and found the antifungal activity achieved at the maximum value when the liquid volume was 100 mL loaded in 250 mL flask (Fig. S8D). In 48 h, the antifungal activity was increased with culture time, and achieved at the maximum value at 48 h (Fig. S8E).

A B


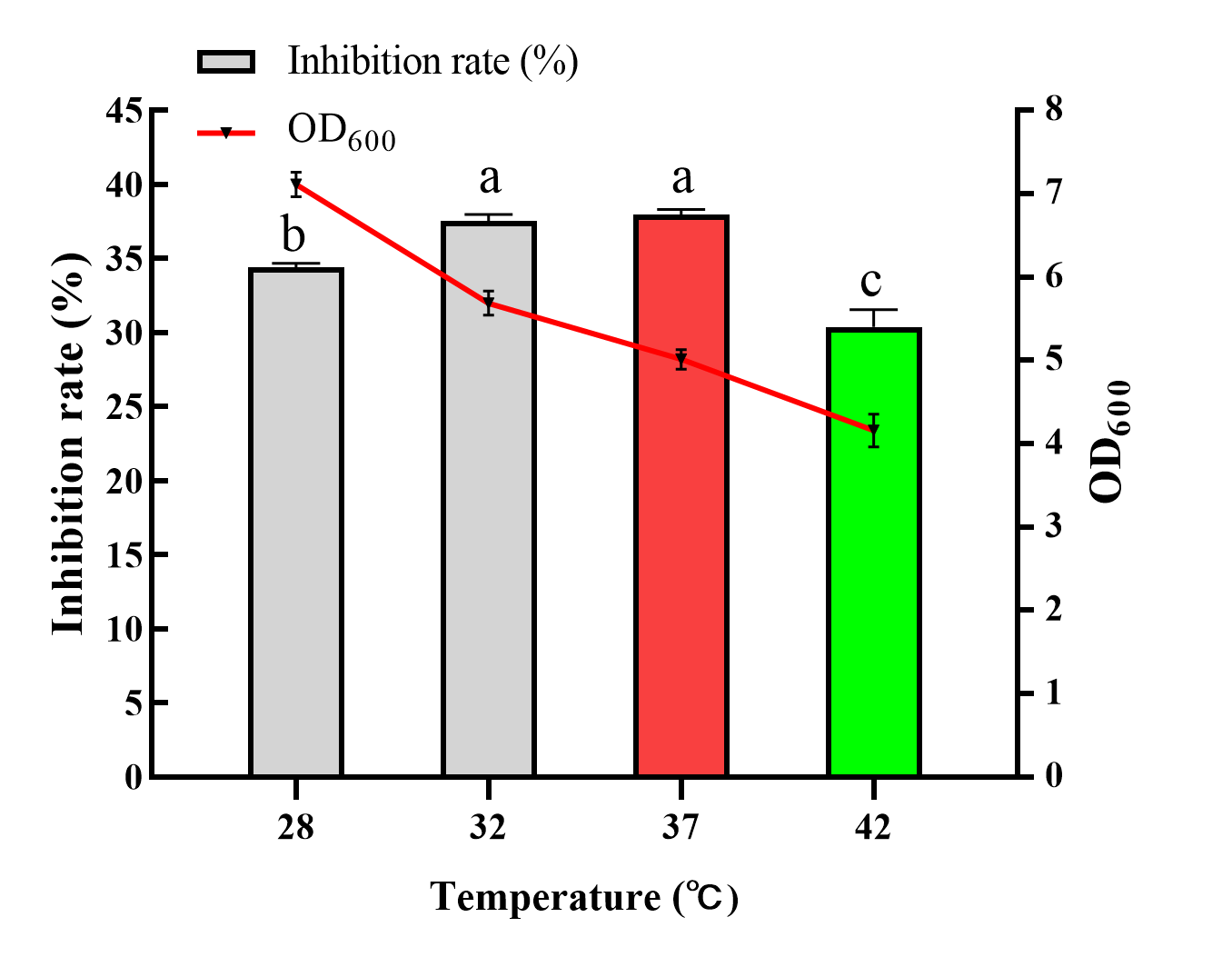

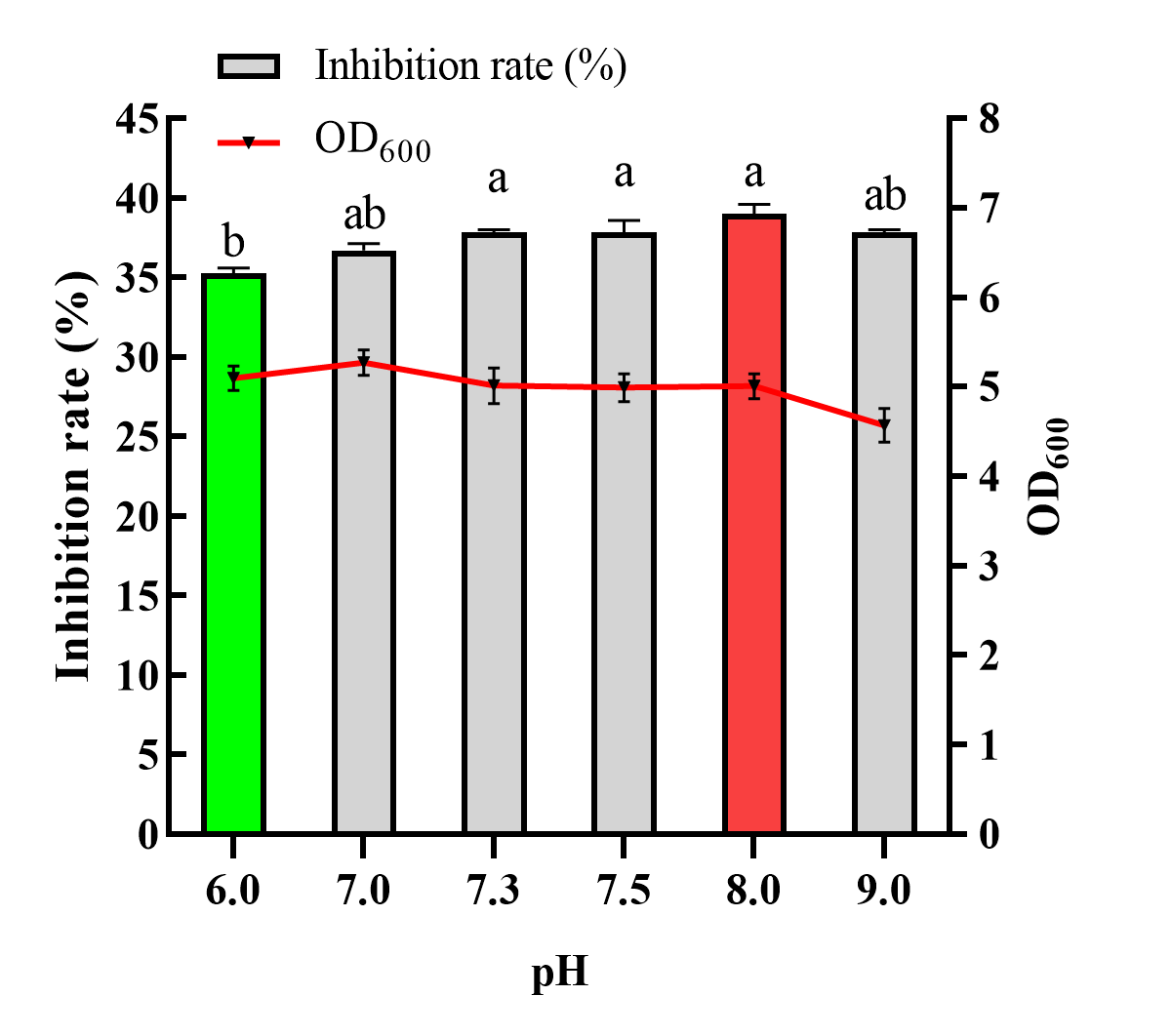


C D


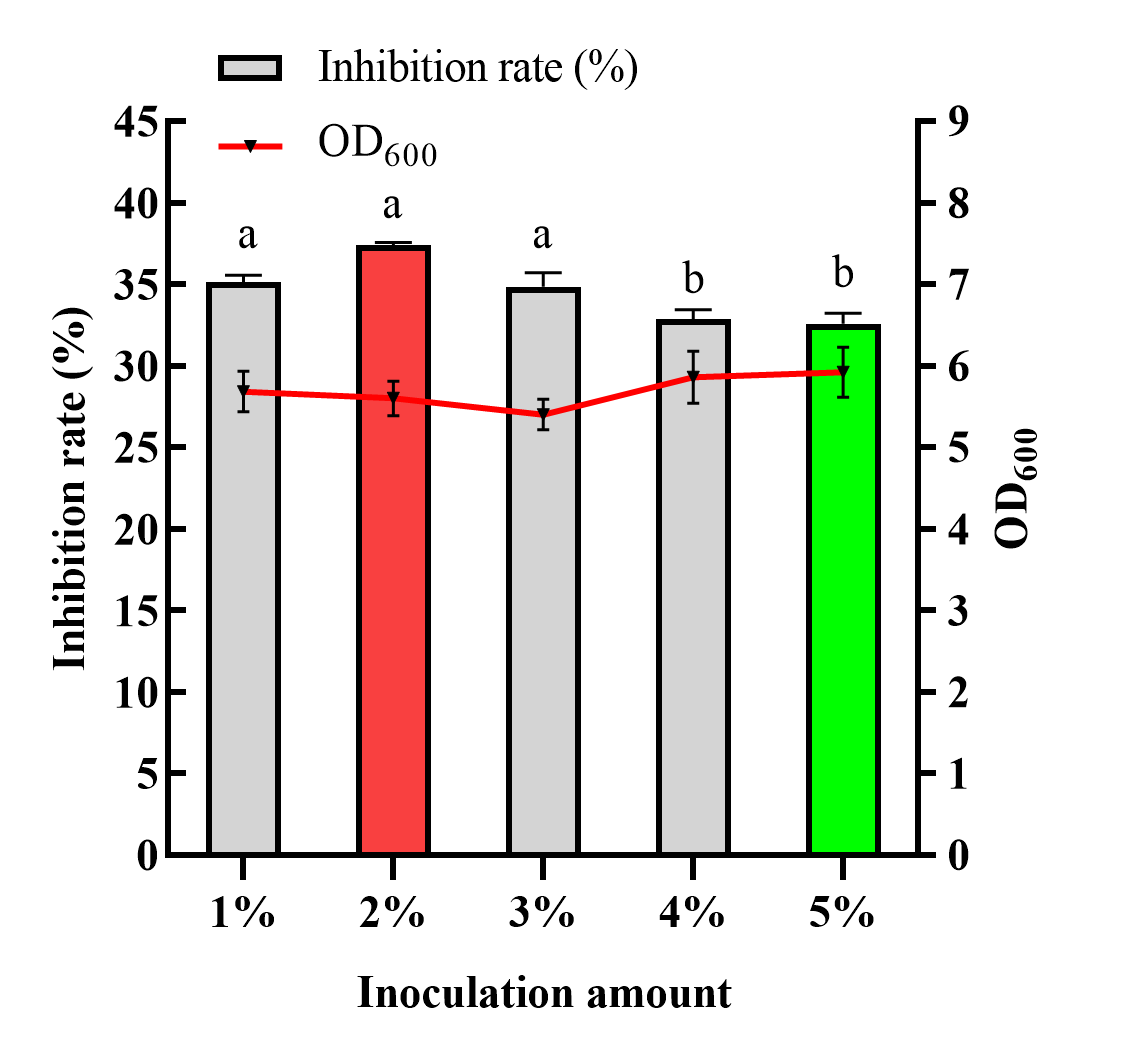

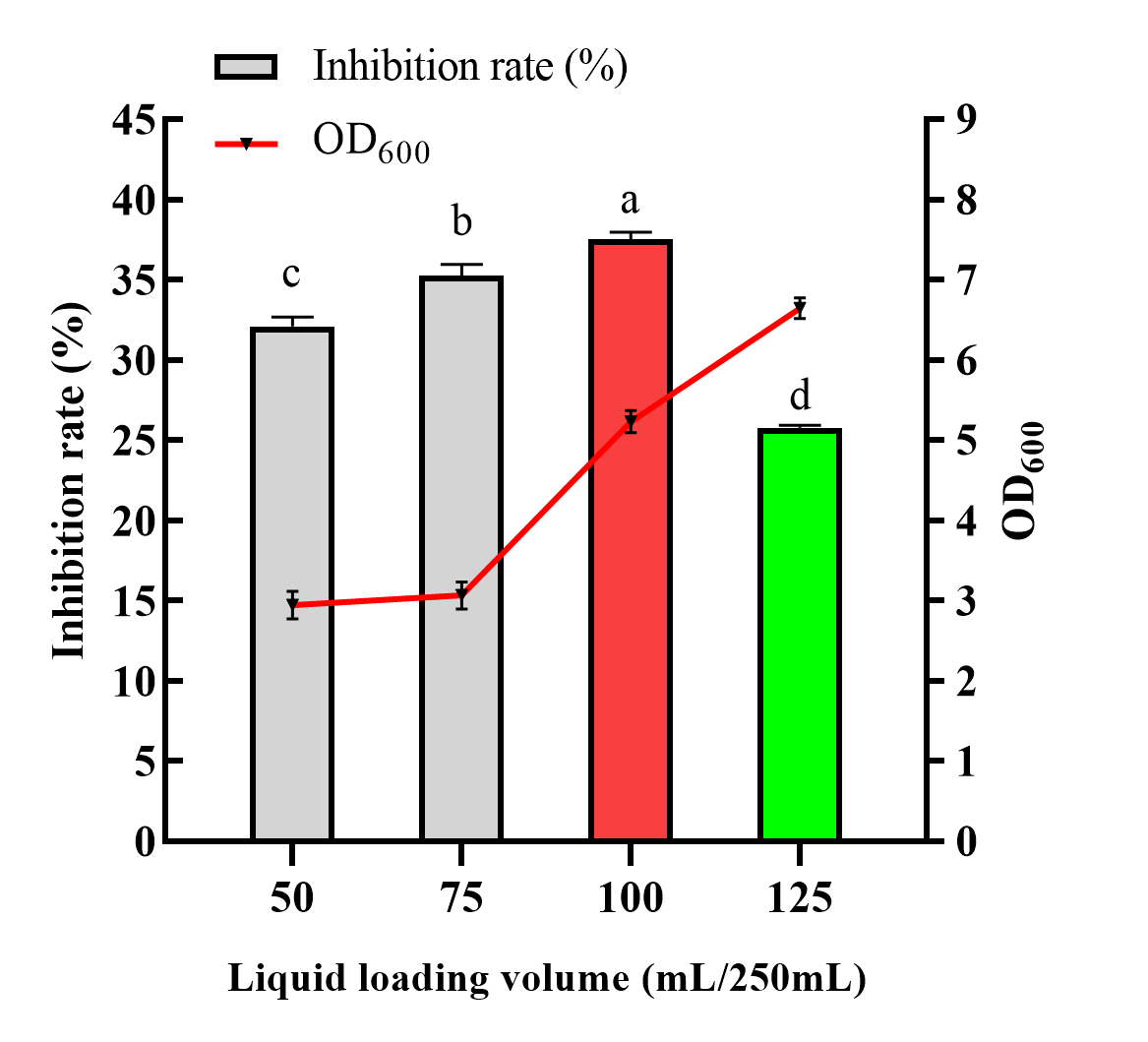


E


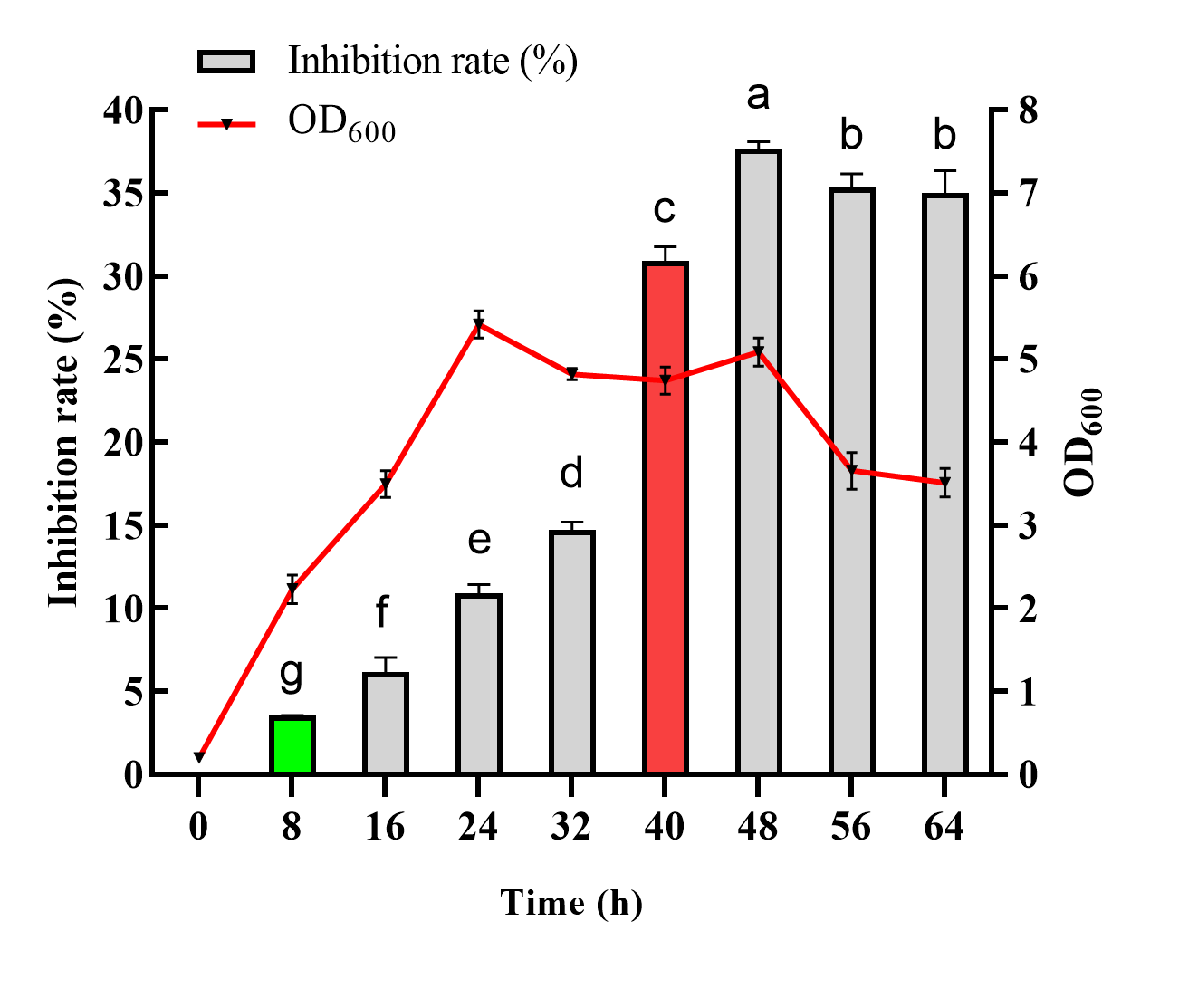


**Fig. S8 Optimization of fermentation conditions for Δ*kin*AΔ*bdh*Δ*dhbF*Δ*rapA*/T2-*sfp* to produce antifungal lipopeptides. A:** Effects of temperature on inhibition rate and biomass (OD_600_) . **B:** Effects of initial pH on inhibition rate and biomass (OD_600_). **C:** Effects of inoculation amount on inhibition rate and biomass (OD_600_). **D:** Effects of liquid loading volume on inhibition rate and biomass (OD_600_). **E:** Effects of fermentation time on inhibition rate and biomass (OD_600_).

**Reference**

Chen, B., Wen, J., Zhao, X., Ding, J., & Qi, G., 2020. Surfactin: A Quorum-Sensing Signal Molecule to Relieve CCR in Bacillus amyloliquefaciens. Front. Microbiol. 11, 631. https://doi.org/10.3389/fmicb.2020.00631.

Fu, Y., Guo, Q., Dong, L., Liu, X., Chen, X., Wang, P., Su, Z., Ma, P., 2022. iTRAQ-based proteomic analysis of Bacillus subtilis strain NCD-2 regulated by PhoPR two-component system: A comparative analysis with transcriptomics revealed the regulation for fengycin production by branched chain amino acids. Microbiol. Res. 260, 127024. https://doi.org/10.1016/j.micres.2022.127024.

Horsburgh, M.J., Moir, A., 1999. Sigma M, an ECF RNA polymerase sigma factor of Bacillus subtilis 168, is essential for growth and survival in high concentrations of salt. Mol. Microbiol. 32(1), 41-50. https://doi.org/10.1046/j.1365-2958.1999.01323.x.

Li, Y., Xia, M., He, P., Yang, Q., Wu, Y., He, P., Ahmed, A., Li, X., Wang, Y., Munir, S., He, Y., 2022. Developing *Penicillium digitatum* Management Strategies on Post-Harvest Citrus Fruits with Metabolic Components and Colonization of *Bacillus subtilis* L1-21. J. Fungi (Basel). 8(1), 80. https://doi.org/10.3390/jof8010080.

Qi, G., Kang, Y., Li, L., Xiao, A., Zhang, S., Wen, Z., Xu, D., Chen, S., 2014. Deletion of meso-2,3-butanediol dehydrogenase gene budC for enhanced D-2,3-butanediol production in Bacillus licheniformis. Biotechnol. Biofuels 7(1), 16. <https://doi.org/10.1186/1754-6834-7-16.>

Yang, R., Lei, S., Xu, X., Jin, H., Sun, H., Zhao, X., Pang, B., Shi, J., 2020. Key elements and regulation strategies of NRPSs for biosynthesis of lipopeptides by Bacillus. Appl. Microbiol. Biotechnol. 104(19), 8077-8087. https://doi.org/10.1007/s00253-020-10801-x.

Zhang, W. J., Guo, P., Liu, M., Yang, B. L., Wang, J. H., & Jiang, J., 2016. Isolation, identification, and optimal cultivation of a marine bacterium antagonistic to Magnaporthe grisea. Genet. Mol. Res. 15(2). https://doi.org/10.4238/gmr.15028646.
